# Synapse Dysfunctions in Multiple Sclerosis

**DOI:** 10.3390/ijms24021639

**Published:** 2023-01-13

**Authors:** Karin Schwarz, Frank Schmitz

**Affiliations:** Department of Neuroanatomy, Institute of Anatomy and Cell Biology, Medical School, Saarland University, 66421 Homburg, Germany

**Keywords:** multiple sclerosis, synapse, astrocyte, microglia, glutamate, ionotropic glutamate receptors, synaptopathy, glutamate excitotoxicity

## Abstract

Multiple sclerosis (MS) is a chronic neuroinflammatory disease of the central nervous system (CNS) affecting nearly three million humans worldwide. In MS, cells of an auto-reactive immune system invade the brain and cause neuroinflammation. Neuroinflammation triggers a complex, multi-faceted harmful process not only in the white matter but also in the grey matter of the brain. In the grey matter, neuroinflammation causes synapse dysfunctions. Synapse dysfunctions in MS occur early and independent from white matter demyelination and are likely correlates of cognitive and mental symptoms in MS. Disturbed synapse/glia interactions and elevated neuroinflammatory signals play a central role. Glutamatergic excitotoxic synapse damage emerges as a major mechanism. We review synapse/glia communication under normal conditions and summarize how this communication becomes malfunctional during neuroinflammation in MS. We discuss mechanisms of how disturbed glia/synapse communication can lead to synapse dysfunctions, signaling dysbalance, and neurodegeneration in MS.

## 1. Primer on Multiple Sclerosis (MS): Multi-Faceted Neuroinflammatory Autoimmune Disease with Pathologies in the White and Grey Matter of the Brain

Multiple sclerosis (MS) is a chronic neuroinflammatory autoimmune disease of the human central nervous system (CNS). Almost three million people worldwide suffer from MS [1]. Among those, young adults (in particular women) are the preferentially affected disease group [2,3,4,5,6,7]. Although the cause of the disease remains elusive, it has become evident that environmental factors and multiple gene loci are risk factors for disease susceptibility [3,8,9,10,11,12,13,14,15,16]. Viral infections, particularly those with the Epstein–Barr virus, have also been proposed to enhance the likelihood of developing MS [17,18,19]. In a longitudinal study, Bjornevik et al. [20] provided evidence that infection with the Epstein–Barr virus is indeed the main trigger for the development of MS, leading to a 32-fold increase in disease susceptibility. In MS, brain-reactive, encephalitogenic T cells (particularly T_H_-cells) from the body periphery invade the brain [21] and induce an auto-destructive immune response that leads to alterations both in the white and grey matter. These auto-reactive T-lymphocytes are the key drivers of the disease [22]. However, also abnormally activated glial cells [23,24] and B-lymphocytes play an important role [22,25,26,27,28,29,30,31]. Long-term depletion of B-lymphocytes by targeting CD20 with monoclonal antibodies can attenuate disease progression in relapsing-remitting but also primary progressive multiple sclerosis [32,33,34,35], emphasizing the role of different components of the immune system in establishing and maintaining the disease.

MS patients suffer from a plethora of clinical symptoms that mirror the sites of lesion. The symptoms include, for example, visual impairments/optic neuritis, central motor paresis, sensory dysfunctions (numbness/paresthesia), and sensory ataxia [36,37,38,39,40,41]. These clinical symptoms are considered “white matter” symptoms resulting from demyelination and axonal damage of the respective fiber tracts in the white matter. In the CNS, the axonal myelin sheath is produced by oligodendrocytes (OL). Oligodendrocyte precursor cells can differentiate into OL and play an important role in the disease course [42]. In the most frequent form of MS, classified as relapsing/remitting multiple sclerosis (RRMS), the disease is characterized by acute inflammatory episodes that improve to some extent before symptoms become progressively chronic and worse with no or only incomplete remission (progressive forms of MS) [16,43,44]. Brain auto-reactive, T-lymphocytes (mainly CD4^+^ T-helper cells) that enter the brain (either via the blood-brain-barrier or passage through the meninges) play a central role in white matter changes and axonal demyelination [16,22,45,46,47]. Auto-antibodies that cross-react with brain epitopes [48,49,50,51,52,53,54] and hyper-activated glial cells [55,56,57] also significantly contribute to the disease. Demyelination and neuro-axonal damage in the white matter have been extensively investigated [16,43,58,59,60,61,62,63,64,65,66] and represent the most appreciated, best-characterized part of MS.

More recently, also alterations in the grey matter of MS patients have been discovered and recognized as an important contributor to the disease [59,67,68,69,70,71,72,73,74]. The novel focus on the grey matter was motivated by the observation that MS patients often show cognitive and psychic symptoms like memory dysfunctions, fatigue, and mood disorders (e.g., depression) [74,75,76,77,78,79,80,81,82,83,84]. These cortical dysfunctions occur even at the early stages of the disease [76,77,85], independent from demyelination in the white matter and are difficult to reconcile with changes only in the white matter. In support of involvement of the grey matter in MS, MRI analyses found lesions in distinct cortical areas, e.g., the hippocampus, temporal cortex, and deep grey matter [70,71,80,84,86,87,88,89,90,91]. In line with the MRI data, analyses of post-mortem brains from MS patients revealed morphological and molecular synapse alterations [66,68,70,71,80,84,86,87,88,89,92,93,94,95]. These data pointed to synapse dysfunctions [66,68,70,71,80,84,86,87,88,89,92,93,94]. Similar alterations were observed in rodent models of multiple sclerosis [70,71,84,93,95,96,97]. These animal models mimic important aspects of the human disease [22,98,99,100] and are important to make the alterations observed in human MS patients accessible for systematic analyses and basic research. A frequently used and well-validated animal model of MS is the experimental autoimmune encephalomyelitis (EAE) mouse model, in which the autoimmune disease is induced by active immunization with an encephalitogenic peptide (e.g., MOG_35–55_) from the myelin oligodendrocyte glycoprotein (MOG) [101,102,103]. In C57BL/6J mice, MOG-induced EAE resembles a model for chronic progressive MS [98,100]. After a defined pre-clinical period, the EAE mice develop characteristic clinical symptoms in a reproducible manner, starting ≈10 days after initial immunization [102,103]. The onset of synaptic changes in this EAE rodent model occurred early before the onset of clinical symptoms and independent of demyelination, arguing that the synaptic changes are not secondary to demyelination but independent or primary events [70,84,87,93].

Neuroinflammation strongly contributes to grey matter changes [69,70,74,85,104,105]. Neuroinflammation involves abnormal glial cell activation and an excessive release of inflammatory cytokines that cause excitotoxic synapse damage [69,106,107,108]. Excitotoxic synapse damage can cause cortical network dysfunctions, cognitive disabilities, neurodegeneration, and neuronal cell death that could lead to irreversible disease progression. Clearly, the pathogenesis of grey matter is complex and not completely understood. However, a general picture of MS-related changes in the grey matter is gradually emerging, which we attempt to summarize in the present review.

## 2. Primer on Brain Synapses: Communication Nano-Machines with Multiple Adjustment “Screws”

The remarkable capabilities of the human brain are enabled by neuronal networks formed by about 100 billion neurons that are connected by more than 100 trillion chemical synapses (10^14^ synapses). Recent evidence revealed that neuronal synapses, the key devices of intercellular communication in the grey matter of the brain, are compromised in MS. Synaptic dysfunction and subsequent neurodegeneration likely account for the cognitive changes and for disease progression in MS. Before reviewing synapse alterations and dysfunctions in MS, we will summarize key aspects of synapse structure, function, and plasticity under healthy conditions. In MS, glutamatergic synaptic signaling is particularly altered. Therefore, we will focus mainly on glutamatergic synapses in this review.

**Figure 1:**
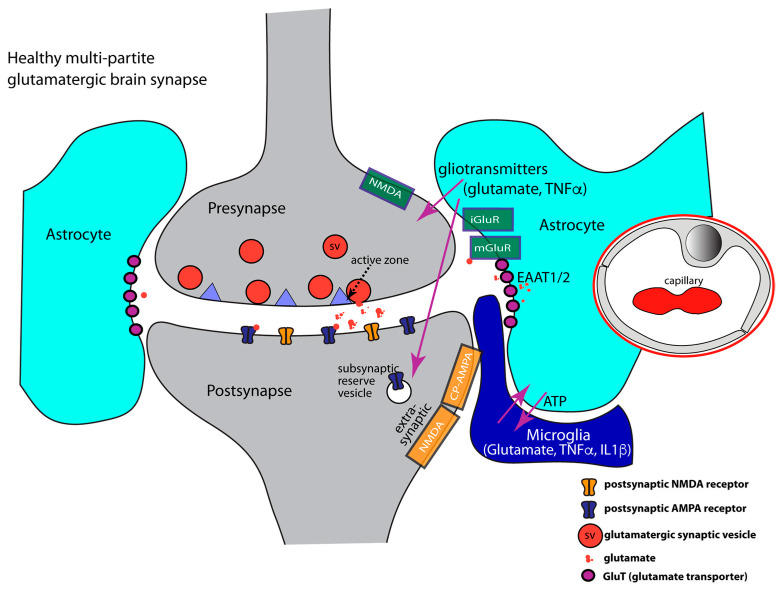
Glutamatergic brain synapses. It schematically depicts the composition of a healthy, glutamatergic tetrapartite brain synapse consisting of pre- and postsynaptic terminals and perisynaptic processes of astrocytes and microglia. At the presynaptic terminal, synaptic communication occurs via exocytosis of glutamatergic synaptic vesicles at the active zone. The active zone is a protein-rich compartment at which the synaptic vesicle fusion machinery is linked by active zone proteins close to voltage-gated Cav-channels. Influx of Ca^2+^ through Cav-channels triggers exocytosis. The postsynaptic membrane contains ionotropic glutamate receptors. AMPA and NMDA receptors are depicted. Perisynaptic processes of astrocytes contain glutamate transporters that remove synaptically released glutamate to prevent spillover of glutamate to neighboring synapses. Perisynaptic processes from astrocytes communicate with microglia processes (magenta arrows). Astrocytes sense synaptic activity via various receptors, including metabotropic glutamate receptors, and modulate synaptic activity via release of “gliotransmitters”, e.g., glutamate, TNFα and IL1β (magenta arrows). Furthermore, astrocytes control cerebral blood flow and the integrity of the blood-brain barrier. An exemplary capillary is shown with endothelial cell contacts sealed by tight junctions. Arrows in magenta show interactions between components of the multipartite synapse. Abbreviations: CP-AMPA, Ca^2+^-permeable AMPA receptors, iGluR, ionotropic glutamate receptor, mGluR, metabotropic glutamate receptor, EAAT, excitatory amino acid transporter.

Neuronal synapses were traditionally considered to be composed of only two basal morphological units, i.e., a presynaptic terminal and a postsynaptic compartment (Figure 1). The presynaptic terminal contains synaptic vesicles that are filled with neurotransmitters. After appropriate stimulation, synaptic vesicles fuse with the presynaptic plasma membrane and release the neurotransmitter contents into the synaptic cleft. Synaptic vesicle fusion is triggered by Ca^2+^ entry through voltage-gated Ca^2+^ (Cav) channels and subsequent activation of members of the synaptotagmin Ca^2+^ sensor protein family [109,110]. Fast synaptic vesicle fusion is mediated by SNARE proteins and preferentially occurs at the active zone (Figure 1). Active zone proteins recruit the vesicle fusion/priming machinery near voltage-gated Cav channels [111,112]. The distance between Cav channels and the vesicle release machinery is relevant for synaptic vesicle release probability [113]. Different types of voltage-gated Ca^2+^ channels endow the synapse with characteristic signaling properties [114,115]. P-/Q-/N-type of Ca^2+^ channels are found in the active zones of most CNS synapses. Retinal and inner ear ribbon synapses contain presynaptic L-type Cav channels [116,117]. Some synapses even contain multiple types of Cav channels [118]. Following synaptic vesicle fusion, retrieval of fused synaptic vesicle membrane and vesicle proteins are recovered via different types of endocytosis [119].

After release into the synaptic cleft, the neurotransmitter (e.g., glutamate) binds to postsynaptic neurotransmitter receptors. The neurotransmitter receptors are enriched in the postsynaptic density (PSD) that is located directly opposite to the active zone. In the PSD, neurotransmitter receptors are immobilized by a dense protein network of scaffold proteins [120,121]. These scaffold proteins include MAGUK family proteins, e.g., PSD-95, SAP-97, SAP-102, in excitatory synapses. Inhibitory synapses contain scaffold proteins such as gephyrin and collybistin [122]. PSD-95 of excitatory synapses is a particularly relevant PSD scaffold protein of excitatory synapses because it links AMPA- and NMDA-type glutamate receptors to each other [120,121]. These glutamate receptors are important for synaptic plasticity and for excitotoxic synapse damage (see below).

The synaptic cleft contains synaptic adhesion protein complexes that align pre- and postsynaptic signaling complexes into functionally connected transsynaptic nanocolumns required for synchronous, efficient information transfer [123,124,125]. An important synaptic adhesion complex consists of presynaptic neurexins and postsynaptic neuroligins and functions as transsynaptic organizers [123,124,126]. Neurexin genes generate large numbers of splice variants relevant for guiding connectivity between distinct, individual neurons. Dysfunctions of neurexin-neuroligin synaptic complexes were correlated with cognitive disturbances and neuropsychiatric diseases [123,124]. Other synaptic adhesion complexes, in part, also interact with the neurexin-neuroligin trans-synaptic adhesion axis [123,126].

## 3. Communication at Glutamatergic Synapses

Excitatory glutamatergic synaptic signaling appears to be strongly enhanced in MS and mouse models of MS [78,84,85,87,127,128,129,130]. Glutamate is the major excitatory neurotransmitter of the central nervous system (CNS) [131,132,133,134] and is of particular relevance in MS. The levels of glutamate in the cerebrospinal fluid (CSF) of MS patients and EAE mice are increased [70,129,135,136,137,138,139,140], pointing to the particular importance of glutamatergic signaling dysfunctions in MS.

Exocytosis of glutamatergic synaptic vesicles at the active zone is the prime mechanism for glutamate release at the synapse. Following presynaptic release, glutamate exerts its action at the postsynapse by binding to different types of glutamate receptors [131,132,133] (Figure 1). Glutamate receptors are classified into ionotropic and metabotropic glutamate receptors (iGluR, mGluR) [133,141]. iGluRs are sub-divided into AMPA-, Kainate, and NMDA-type receptors based on their molecular composition, physiological properties, and preferential agonists. For fast synaptic transmission, the postsynapse mainly employs iGluRs. All iGluR receptors are composed of several subunits. In the CNS, 2-Amino-3-(3-hydroxy-5-methylisooxazol-4-yl)proprionate (AMPA) receptors are the most abundant glutamate iGluRs. AMPA receptors assemble from four different subunits (GluA1-GluA4). These form homo- or hetero-tetramers [133,142,143]. Glutamate-gated opening of AMPA receptors depolarizes the postsynaptic compartment. AMPA receptors are permeable to Na^+^, K^+^ and, depending upon subunit composition, also to Ca^2+^. If the GluA2 subunit is absent from AMPA receptors, the resulting AMPA channels are permeable to Ca^2+^ [133,142]. AMPA receptors containing GluA2 are not Ca^2+^ permeable. N-methyl-D-aspartate (NMDA)-type iGluRs also play an important role for synaptopathy in MS. NMDA receptors are hetero-tetramers that consist of GluN1, GluN2 (GluN2A, GluN2B, GluN2C, GluN2D), and GluN3(A,B) subunits [144,145]. Two GluN1 subunits combine with two GluN2 or GluN3 subunits to form the NMDA channel. AMPA and NMDA receptors functionally interact. Strong depolarization of the postsynaptic compartment obtained by many AMPA-channel openings relieves the Mg^2+^ block of NMDA receptors and enables the opening of NMDA receptor channels [141,145,146]. Only strong depolarizations that typically result from multiple simultaneous presynaptic vesicle fusion events lift the block of NMDA glutamate receptors by expulsing Mg^2+^ from the channel pore. The influx of Ca^2+^ through the NMDA receptor induces early and late phases of LTP (Long-Term Potentiation) through CaMKII (Ca^2+^/calmodulin-dependent protein kinase II) and cAMP/PKA/pCREB-dependent mechanisms [147,148,149,150,151,152,153,154,155,156]. Influx of Ca^2+^ also triggers increased surface expression of AMPA receptors via fusion of AMPA receptor-containing subsynaptic vesicles with the postsynaptic plasma membrane [147,157,158]. Elevated surface expression of AMPA receptors increases the efficacy of this individual synapse at which the NMDA receptor was activated and makes it more sensitive to the subsequent release of glutamate by the connected presynaptic terminal. The adjustment of synaptic efficacy based on previous activity is part of a phenomenon called “synaptic plasticity”. It is considered the basis for learning, memory, and experience-based behavior [159]. Heavily used synapses become more efficient by this mechanism. Vice versa, the efficacy of less active synapses decreases. These positive feedback mechanisms belong to the Hebbian-type of synaptic plasticity and include short-term and long-term effects [147,157,160]. The Ca^2+^ permeability of iGluRs (GluA2-lacking AMPA- and NMDA receptors) is central to this process [142,161,162,163]. Further Ca^2+^-dependent events also support synaptic plasticity. Ca^2+^ regulates the metabolic activity of mitochondria in order to synchronize energy production with synaptic activity [164,165,166]. Ca^2+^ also controls intracellular signaling cascades, such as the Ca^2+^/Calmodulin/CaMKII system and the Ras/Raf/MAP-kinase pathway that control the activity of phosphorylation-regulated transcription factors (e.g., CREB, NF-kB). These transcription factors control transcriptional programs needed for the long-term remodeling of synaptic components (e.g., dendritic spines) and/or neuronal survival (e.g., secretion of BDNF [167]). Pre-synaptic mechanisms can also contribute to synaptic plasticity [112,150,168]. Presynaptic mechanisms include modulation of synaptic vesicle release probability, e.g., by modification of active zone components/Cav-channel number/distance/opening properties [112,113]. A second form of synaptic plasticity, homeostatic synaptic plasticity (HSP), prevents over-activation/saturation of active synapses and complete silencing of less frequently used synapses [169,170,171,172,173,174]. HSP is based on negative-feedback mechanisms that lead to counter-acting, compensatory adjustments, and a re-setting of the synaptic signaling range. HSP prevents saturation and unresponsiveness of synaptic connections and maintains synaptic activity in a functional range [169,170]. Interestingly, synaptic plasticity and adaptational changes of synapses are strongly influenced by inflammatory cytokines, such as TNFα and IL1β.

## 4. A More Extended View on Brain Synapses: Contribution of Glial Cells

In the brain, pro-inflammatory cytokines, such as TNFα and IL1β, are physiologically secreted in small amounts by glial cells, mainly by astrocytes and microglia. These glial cells establish close contacts with synapses and modulate synaptic communication. Glia-synapse interactions are important to adjust synaptic activity under normal conditions but are also relevant for synaptopathy as it occurs in MS.

## 5. Primer on Astrocytes: A Network of Guardians of Brain Homeostasis with Strong Impact on Synapses

The human brain contains about 86 billions of neurons and a similar number (85 billions) of glial cells [175]. Within the population of glial cells, astrocytes are the most abundant type of glial cells in the human brain. Astrocytes are multi-branched stellate cells that intensively communicate with each other via gap junctions [176,177,178]. Astrocytes also establish close contact with neurons. At a functional level, astrocytes execute many pivotal homeostatic functions. Some important functions include the maintenance of extracellular ion and fluid balance, provision of metabolites (e.g., glutamine, see below) to neurons, control of blood flow, and maintenance of the blood-brain barrier [176,179,180,181,182,183,184,185]. Astrocytes are functionally very diverse, as also confirmed by single-cell sequencing [178]. Based on the molecular heterogeneity, a nomenclature has been proposed for astrocytes in which resting astrocytes and various types of “reactive” astrocytes have been discriminated [178].

Astrocytes are also relevant for synapses in the brain. Astrocytes form processes that wrap around synapses, thus establishing close perisynaptic contacts with pre- and postsynaptic compartments [83,177,186,187,188] (Figure 1). A single astrocyte can ensheath more than 100,000 synapses [189,190,191]. The degree of ensheathment of synapses by perisynaptic processes varies between brain regions [192,193]. Perisynaptic processes of astrocytes have a strong impact on synapse function. Therefore, astrocytes have been considered an integral part of chemical brain synapses, which has been coined “tripartite” synapse [194]. The “tripartite” synapse consists of presynapse, postsynapse, and perisynaptic astrocytic process according to this terminology [177,190,195,196,197] (Figure 1). Synapse-associated perisynaptic processes of astrocytes, sometimes also called “astrocytic cradle“, are important for several aspects of synaptic function [83,177,194,197]:

(1) Astrocytes play an important role in synapse development [177,198]. Astrocytes secrete synaptogenic factors that promote synapse formation and maturation, e.g., synapse organizing molecules, such as thrombospondin, hevin, or trophic factors that promote presynaptic differentiation [177,199,200,201,202,203,204,205,206,207,208]. Astrocytes also secrete glypicans that increase the surface expression of postsynaptic AMPA receptors [209,210].
(2) At glutamatergic synapses, the perisynaptic processes of astrocytes contribute to the uptake of synaptically released glutamate [186,189,192,211,212,213,214,215,216,217,218,219]. Glutamate is taken up by various glutamate transporters [220,221,222]. Glutamate transporters (GluTs), also called excitatory amino acid transporters (EAATs), belong to the solute carrier 1 family (SLC1). Five sodium-dependent glutamate transporters (GluT) of the SLC1 family have been cloned: EAAT1/GLAST1, GLT1/EAAT2, EAAT3/EAAC1, EAAT4, and EAAT5/SLC1A7 [221,223,224,225,226]. GluTs have been localized to different localizations at the synapse. In general, GluTs are present either in the plasma membrane of the presynaptic terminal or in the plasma membrane of perisynaptic astroglial processes [222,227,228,229,230,231,232]. In this way, presynaptic neuronal and glial (astrocytic) glutamate uptake mechanisms collaborate to maintain low resting concentrations of extracellular glutamate and to prevent excitatory over-stimulation/excitatory synapse damage [84,190,216,220,233].

**Figure 2:**
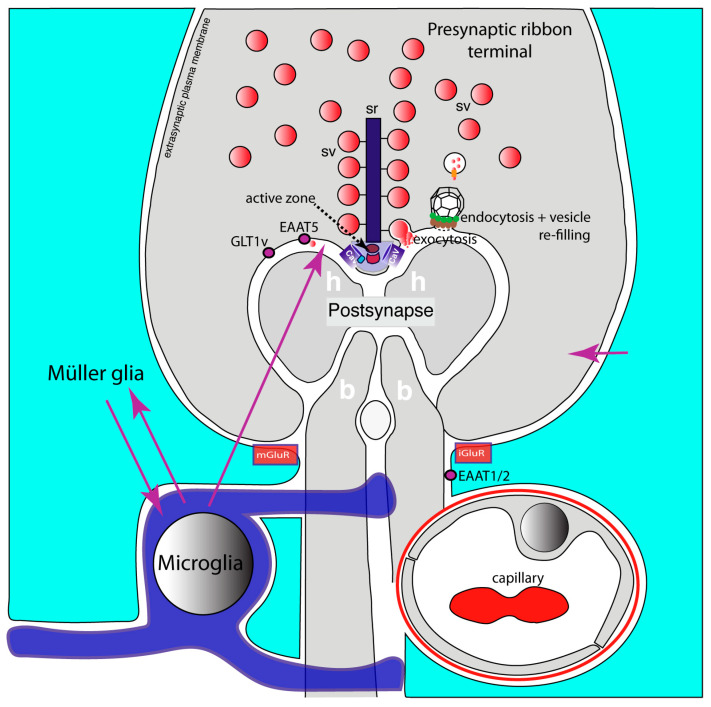
Glutamatergic retinal ribbon synapses. It schematically depicts a glutamatergic ribbon synapse of the retina. A photoreceptor ribbon synapse is shown. The functional and molecular composition of the presynaptic ribbon terminal is like that of the brain synapse shown in Figure 1. However, the presynaptic terminals of ribbon synapses possess eponymous synaptic ribbons that provide the active zone with additional synaptic vesicles to enable continuous synaptic transmission at this synapse. The active zone is the site at which exocytosis of synaptic vesicles occurs close to voltage-gated L-type Cav-channels. Endocytosis of fused vesicle membrane occurs in the periactive zone, followed by a refilling process of endocytosed vesicles with glutamate. The postsynapse is composed of the dendrites of horizontal and bipolar cells. The postsynaptic dendrites contain ionotropic and metabotropic glutamate receptors (not shown). Müller glial cells form perisynaptic processes that are enriched in glutamate transporters (EAAT1, EAAT2). The presynaptic terminal contains additional glutamate transporters (GLT1v, EAAT5) in the periactive zone. Retinal ramified microglial cells frequently contact synapses and likely communicate with Müller glial cells (magenta arrows). Retinal ribbon synapses are very sensitive to neuroinflammatory changes. Neuroinflammatory changes strongly affect presynaptic terminal functions (for details, see text). Active zone composition, presynaptic Ca^2+^ homeostasis and exo- and endocytic vesicle cycling in these synapses are disturbed in EAE. The underlying mechanisms are not yet fully elucidated but might involve simar pathways as described for brain synapses (see text). Arrows in magenta indicate possible interactions between Müller cells, microglia and the photoreceptor synapse. Abbreviations: sv, synaptic vesicles; sr, synaptic ribbon; Cav, voltage-gated Ca^2+^-channel; mGluR, metabotropic glutamate receptor at Müller glial cells; iGluR, ionotropic glutamate receptor at Müller glial cells; h, horizontal cell postsynaptic dendrite; b, bipolar cell postsynaptic dendrite; GLT1v, glutamate transporter 1 splice variant; EAAT1/2, excitatory amino acid transporter 1/2.

GLT1/EAAT2 accounts for most of the glutamate uptake in the brain [222,231,234]. GLT1/EAAT1 is expressed predominantly, although not exclusively, in astrocytes [222,231,234]. GLT1/EAAT2 is enriched in perisynaptic astrocytic processes [187,216,230]. Splice variants of GLT1/EAAT1 and EAAT5 are localized in presynaptic terminals close to the presynaptic release sites [235,236,237,238]. Moreover, GLAST/EAAT1 is localized to astroglia in perisynaptic processes. Glutamatergic ribbon synapses are organized in a similar manner (Figure 2). Ribbon synapses of the retina are strongly affected in mouse models of MS [239,240,241], and their early dysfunction might contribute to optic neuritis, a frequent symptom in MS [242]. In the retina, EAAT1/2 is mostly found in the perisynaptic processes of Müller glial cells (Figure 2) [243,244,245,246,247,248,249,250,251]. At a perisynaptic location, astrocytic GluTs are strategically well placed to remove glutamate spillovers and to prevent crosstalk to neighboring synapses. In astrocytes or astrocyte-related radial glial cells (Müller cells of the retina, Bergmann glia of the cerebellum), glutamate is metabolized into glutamine via glutamine synthetase. Glutamine, a metabolically inert form of glutamate, is re-provided to the neuron in order to replenish the neuronal glutamate stocks (glutamate-glutamine cycle [132,222,252]. EAAT5 has been localized in presynaptic terminals of retinal photoreceptors and retinal bipolar cells close to presynaptic release sites [228,253,254,255].

Of note, glutamate transporters (GluTs) can also revert the transport direction of glutamate and secrete glutamate. This can occur during pathological conditions/diseases. The physiological basis for this phenomenon is the fact that GluTs are secondary active transporters that depend upon the transcellular Na^+^ gradient [221]. In this electrogenic transport, one glutamate molecule is co-transported with 3 Na^+^ and 1 H^+^. In the regular “forward” mode, this import is coupled with counter-transport (export) of a K^+^ ion into the extracellular space. Disease conditions that dissipate the electrochemical Na^+^ gradient can drive glutamate transport into the opposite direction, i.e., release of glutamate into the extracellular space (reverse mode of transporter activity [256,257]). Reverse efflux of glutamate through GluTs happens during ischemia and inflammation [257,258,259]. Such a mechanism could also be relevant for glutamate excitotoxicity in MS (see below). Further mechanisms that could contribute to the elevation of extrasynaptic glutamate include glutamate release through the cystine-glutamate antiporter xCT (cystine/glutamate antiporter) [260,261], release through bestrophin-1 anion channel [262,263,264,265], TREK-1 channels [264,265], and volume-regulated anion channels/volume-sensitive organic anion channels (VRACs/VSOACs) [266,267,268]. Moreover, vesicular release of glutamate by astrocytes and microglia could lead to elevated levels of extrasynaptic glutamate and contribute to glutamate excitotoxicity in MS (see below).

(3) Perisynaptic astrocytes possess different types of neurotransmitter receptors, e.g., metabotropic glutamate receptors (mGluR2, mGluR3, mGluR5), AMPA-type ionotropic glutamate receptors, GABA receptors, and purinoreceptors to sense synaptic activity [177,196,197,269,270,271,272,273,274]. Astrocytes are capable of secreting TNFα and also possess TNFα receptors that serve autocrine effects. TNFα receptors are also important for communication with microglia [82,162,177,275,276,277] and neurons [162,163,173,278,279,280].

Activation of astrocytic neurotransmitter receptors leads to changes in intracellular Ca^2+^ in astrocytes and to the release of “gliotransmitters” [195,208,272,281,282,283,284,285,286]. The source of these intracellular Ca^2+^ changes is controversially discussed [287,288]. Gliotransmitters are neuroactive molecules, such as glutamate, ATP/adenosine, GABA, NPY, D-serine (a co-agonist of NMDA-type of glutamate receptors), IL1β, and TNF-α [286]. Astrocytes release synapse-active components that affect synaptic performance [208,218,283,284,285,289]. Gliotransmitters released by astrocytes influence pre- and postsynaptic functions [193,195,276,278,290,291]. Important signaling cascades have been identified. Glutamatergic synapses that show only little activity secrete less glutamate. Decreased levels of synaptically released glutamate are sensed by neurotransmitter receptors of perisynaptic astrocytes in hippocampal synapses and induce secretion of TNFα (by astrocytes and microglia via astrocyte/microglia communication). These elevated, non-toxic levels of TNFα (≈100 picomolar TNFα) enhance pre- and postsynaptic glutamatergic signaling [162,163,173,278,292,293,294]. TNFα induces glutamate release by astrocytes that, in turn, stimulate presynaptic glutamate release at hippocampal synapses (i.e., entorhinal cortex/dentate gyrus synapses) via binding to presynaptic NMDA receptors [162,163,269,275,277,278,290,295]. Of note, the effect of astrocytic glutamate on presynaptic release depends on the expression of distinct neurotransmitter receptors. The binding of glutamate to metabotropic glutamate receptors (mGluR2/3) at the presynapse was reported to inhibit glutamate release from the presynaptic terminal [296,297,298]. At the postsynapse, glial TNFα leads to an increased surface expression of AMPA receptors to scale up synaptic activity and synaptic responsiveness [162,163]. In conclusion, the pro-inflammatory cytokine TNFα at physiological concentrations is an important positive regulator of synaptic activity in the healthy brain. Modulation of TNFα secretion in this physiological range serves homeostatic scaling of synapse activity (HSP, see above). Typically, astrocyte-mediated modulation of synaptic transmission occurs at a slower timescale as fast synaptic communication between the pre- and postsynaptic compartment due to the signal integration in the glial compartment [197,272,278]. At the systems level, physiologically elevated levels of pro-inflammatory cytokines have been shown to be important for memory formation in freely moving animals reflecting their effect on synapses also in-situ [299].

Astrocytes establish intimate contacts with microglia at synapses and exchange important mutual signals with microglia. Astrocytes provide microglia with cues on synaptic activity. Microglia vice versa, provide signals for the differentiation of astrocytes into distinct sub-types, i.e., either into a beneficial, homeostatic, and activity-maintaining sub-type (initially denoted as “A2” astrocytes [217,284,300]) or into a neurotoxic, neuroinflammatory subtype (“A1” astrocytes [208]) whose activity is detrimental to the CNS [208,217,284,300]. C1q, IL-1α, and TNFα promote differentiation of resting astrocytes into the A1 neuro-destructive state [208], whereas co-stimulation with TNFα and IL1β promotes differentiation towards the neuro-supportive A2 phenotype in astrocytes [217,284,300,301].

As mentioned, the A1/A2 dichotomy of astrocytes is too simplified based on single-cell sequencing data but still useful as a simplified working model. Transcript analyses revealed markers common to all reactive astrocytes (e.g., GFAP [178,302]). The expression of complement protein C3 is a marker for inflammatory “A1” astrocytes [178,208,284,303,304]. Complement proteins were found to be highly elevated in the brain of MS patients, particularly in cortical grey matter lesions [305,306,307,308]. The complement system is likely involved in synapse dysfunctions in the MS brain based on its well-known function of synapse removal during brain development [309,310]. Astrocyte-microglia interactions are highly relevant for neuroinflammatory disease in multiple sclerosis and the resulting synaptic changes (see below).

## 6. Primer on Microglia: Never-Resting Brain “Police” with the Mission to Survey and to Take (Strong) Action

Microglia are the main resident immune cells of the brain, comprising ≈10% of total CNS brain cells with some regional differences [311,312,313,314,315]. Microglial cells develop from monocyte-like precursor cells of the bone marrow and yolk sac [316,317]. During embryonic development, they invade the brain, in which they mature and proliferate [216,318,319]. Cell surface markers, such as TMEM119, allow to discriminate microglia from blood-borne macrophages invading from capillaries [320,321]. A few years ago, microglia were believed to be active only during disease conditions. Recent investigations demonstrated that microglia perform important functions already in the healthy, non-injured brain. The range of functions performed by microglia, both in the healthy and injured brain, is broad [216,285,322,323,324].

Morphologically, microglia are very diverse and dynamic [325]. In the healthy brain, microglial cells often display a highly ramified morphology with many processes. Ramified microglia were previously considered “resting”, i.e., inactive. This old view is not correct. Novel technologies, particularly live imaging analyses with genetically engineered fluorescent microglia, demonstrated that “resting” ramified microglial cells are indeed highly active already under physiological conditions in the healthy brain [322,326,327,328,329,330,331]. Live imaging experiments with genetically tagged microglia revealed that microglial processes are highly mobile and frequently expand and retract [322,326]. These processes continuously scan and monitor the extracellular environment of the CNS for a broad range of signals [216,285,322,323]. Thus, the term “resting” microglia should be replaced by the term “surveying” microglia [323].

Live-Imaging with these genetically engineered mice possessing fluorescent microglia revealed that ramified microglia use their processes to contact synapses [322,326]. The soma of the microglia typically stays stationary in this process, whereas the processes expand and retract continuously. A sub-type of ramified microglia that is positive for a 5D4 keratan-epitope and rich in IL1β is particularly active in this process [332]. The close relationship between microglial processes to both pre- and postsynaptic compartments, as well as to perisynaptic astrocytes has been referred to as tetra-partite synapse [285,333].

The interaction between ramified microglia processes and synapses is complex and depends upon synaptic activity [327,328]. Synapses and microglial synaptic processes influence each other in a mutual manner. On the one hand, synaptic activity regulates the contact properties between microglial processes and synapses (e.g., process motility, duration, and frequency of synaptic contacts) in a differential manner [334]. On the other hand, ramified microglia signal back to synapses and lead to changes in glutamatergic synaptic transmission [276,334]. The contact between ramified microglial processes and synapses was reported to increase synaptic activity [335]. This feedback from microglia to synapses is predominantly indirect and mediated via astrocytes that secrete gliotransmitters [276,335]. Similarly, synaptic activity also influences the motility of microglial processes in the retina [336]. In the retina, glutamatergic neurotransmission enhances microglia process motility, whereas GABAergic transmission inhibits microglial process motility [336].

The precise mechanisms of synapse-microglia interactions are complex and not fully understood. As mentioned, the effects of synaptically released glutamate on microglia are likely indirect because ramified surveying microglia do not express glutamate receptors (in contrast to “activated” microglia, see below). In the retina, the neurotransmitter effects of glutamate on microglia motility were reported to be mediated via ATP release from Müller glial cells/astrocytes [336]. Thus, synaptic neurotransmitter release likely does not signal directly to ramified microglial cells but indirectly via signals from perisynaptic astrocytes [326,335,336,337,338]. ATP binds to microglial ionotropic P2X7 receptors to induce the release of IL1β [339]. IL1β is a powerful modulator of synapse function (see below). ATP can also bind to microglial metabotropic purinergic receptors (P2Y12/13) that are relevant for chemotactic guiding of microglia [340,341,342]. Released ATP can also directly affect the postsynaptic terminal by activation of postsynaptic P2X receptors [343,344].

Of note, ramified microglia secrete trophic factors (e.g., BDNF, NGF, FGF, and IGF-1) that promote synapse function and synaptic plasticity [167,216,330,333,342,345,346,347,348,349].

## 7. Multivalent Microglia: Potentiator of Inflammatory Signals with Strong Impact on Synapses

Any kind of homeostatic disturbance in the brain can activate microglia and lead to a transformation into a reactive, particularly alerted state [216,285,318,319,323]. Microglia activation is often associated with de-ramification or even loss of microglial processes. A process-lacking amoeboid shape promotes the movement of the activated microglia towards the area in which a potential threat has been detected. The character and degree of microglia activation differ broadly [216,285,323,326,327,328,336,350]. Two extreme forms of activated microglia sub-types have been previously discriminated and denoted as M1- and M2-microglia [284,323,351,352,353]. “M1”-type microglia are pro-inflammatory and neurotoxic with low phagocytic activity. They show surface expression of MHC-II proteins, CD11b, CD16, CD68, TREM2, and release large quantities of glutamate (and glutamate-like toxic kynurenines) as well as pro-inflammatory cytokines, such as IL1β, TNFα, IL-6, IL-12, and IFNγ [216,284,323,342,354,355]. “M2”-type microglia are anti-inflammatory, neuroprotective with high phagocytic activity, express distinct surface markers such as CD163 and CD206, and secrete IGF-1 and TGF-β [216,284,323,342,354,355]. Single-cell sequencing revealed that the classification of activated microglia in only two classes is simplified. Activated microglia are functionally more diverse [302,356,357]. The dichotomic M1/M2 category still serves as a simplified model.

Microglial cells are equipped with various receptors for detecting homeostasis-threatening signals [358,359,360,361,362,363,364]. Large amounts of ATP released from damaged or dead cells are a strong attracting and activation signal for microglia [285,326,365,366]. Complement proteins (e.g., complement proteins C1, C3) are also detected by microglial cells via cell surface complement receptors [177,185,285,309,329]. During development, complement proteins tag excess or dysfunctional synapses and mark these for subsequent elimination by microglia, a process called “synapse stripping”. Synapse stripping is important for the development and refinement of functional neuronal circuits during development [216,309,329]. In the mature brain, less active synapses also contain increased levels of C1q that promote their removal by microglial cells, thus allowing activity-dependent refinement of neuronal circuits in the healthy, postnatal brain [327,328,329,345,367,368,369]. Astrocytes are also involved in synapse stripping of silenced or dysfunctional synapses together with the microglia [177,370].

As mentioned, the activation of microglia can be strong, particularly if the inflammatory event stays unresolved and remains to continuously activate the microglia. Under these conditions, i.e., when microglia are strongly and permanently activated, microglia become harmful to the host brain and promote disease progression/aggravation by secreting large amounts of reactive oxygen species and biologically active cytokines that cause neuroinflammation [216,284,285,348,353,371,372]. Inflammatory over-activation of microglia, as it occurs in active, progressive MS, can lead to significantly elevated release of the pro-inflammatory cytokines, e.g., TNFα, IL1β [70,84,373], that adversely affect synapse functions (see below). As mentioned, microglia also communicate with astrocytes and influence the differentiation of astrocytes. Pathologically activated microglia of the “M1” subtype activate astrocytes towards a harmful “A1” sub-type by the secretion of IL1α, TNFα, and C1q [162,177,208,216,276]. A1 astrocytes, together with the activated M1 microglia, represent the major source of the pro-inflammatory cytokines IL1β and TNFα in the brain [69,162,177,216,276,284]. The large amount of secreted IL1β and TNFα can lead to synapse damage in MS by multiple mechanisms and produce the observed grey matter dysfunctions in MS (see below).

## 8. Neuroinflammation-Induced Synapse Dysfunctions in MS

The levels of the pro-inflammatory cytokines TNFα and IL1β can severely increase in MS, particularly in the active, progressive stages of MS [69,70,78,84,85,128,373,374,375,376,377]. During persistent strong inflammation, activated microglia potentiate inflammatory signals leading to excessive pathological TNFα values up to the mM range [275,278]. A growing body of evidence indicates that the highly elevated levels of these pro-inflammatory cytokines (TNFα, IL1β), as it can occur in MS [378,379], are the main reason for early dysfunctions of synaptic transmission. These lead to glutamatergic excitotoxicity, neurodegeneration, and ultimately, neuronal cell death. Several mechanisms contribute to neuroinflammation-induced glutamatergic synapse dysfunction and glutamatergic excitotoxicity.

**Figure 3:**
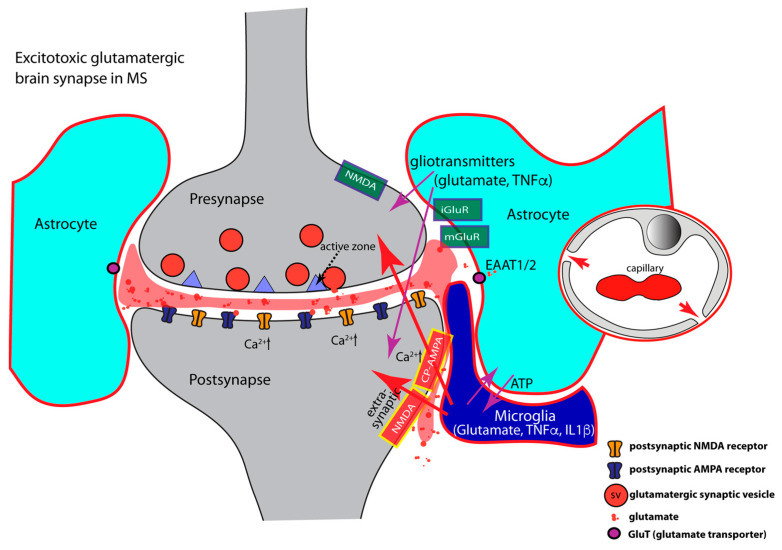
Schematically depicts a brain synapse, as shown in Figure 1, but under neuroinflammatory conditions as in multiple sclerosis (MS). Perisynaptic astrocytes and microglia are strongly activated (encircled in red) and secrete large amounts of inflammatory cytokines. Inflammatory cytokines produce glutamatergic excitotoxicity (see text). Neuroinflammation leads to decreased expression of astrocytic glutamate transporters and to elevated levels of extracellular glutamate (colored in red). Increased extracellular glutamate binds to extrasynaptic glutamate receptors. This initiates a series of deleterious events at the synapse consisting of structural synapse damage, mitochondrial dysfunctions and transcriptional down-regulation of neuroprotective pathways. Synaptic excitotoxicity is further aggravated by increased incorporation of AMPA and NMDA receptors into the postsynaptic membrane, together with a concomitant decrease of inhibitory synaptic transmission (not shown). During neuroinflammation, the integrity of the blood-brain-barrier is compromised and inter-endothelial cell contacts become leaky (indicated by red arrows in the depicted capillary) allowing the entry of blood-borne immune cells into the CNS. Arrows in magenta show interactions between components of the multipartite synapse. Arrows in red denote pathologically activated signaling events during neuroinflammation. Abbreviations: CP-AMPA, Ca^2+^-permeable AMPA receptors; iGluR, ionotropic glutamate receptor; mGluR, metabotropic glutamate receptor; EAAT, excitatory amino acid transporter.

(1) TNFα regulates AMPA- and GABA- receptor trafficking in an antagonistic manner. The highly elevated levels of inflammatory cytokines released by activated microglia, astrocytes, and inflammatory CD3^+^ T-cells in MS, inhibit the expression of glial glutamate transporters (EAAT1/2), resulting in a decreased clearance of glutamate from the synaptic cleft [85,128,129,380,381,382,383,384,385] (Figure 3). The decreased glutamate clearance results in increased levels of extrasynaptic glutamate. Extrasynaptic glutamate binds to extrasynaptic glutamate receptors, including Ca^2+^-permeable NMDA receptors and Ca^2+^-permeable AMPA receptors. Stimulation of these extrasynaptic glutamate receptors is considered the central mechanism causing glutamate excitotoxicity, neurodegeneration, and neuronal cell death [386,387,388,389]. Many of these mechanisms involve elevated levels of Ca^2+^. Extrasynaptic NMDA receptor activation will trigger a deleterious signaling cascade that includes structural degeneration of the synapse, mitochondrial damage, and transcriptional shut-off of neuroprotective pathways [386,387,388,389]. Paradoxically, increased extrasynaptic glutamate can further inhibit the expression of astrocytic glutamate transporters [390], thus fostering a vicious cycle that leads to glutamate excitotoxicity.
(2) Inflammatory cytokines (TNFα, IL1β) induce an increased surface expression of AMPA receptors [85,127,129,162,163,391,392] (Figure 3). Increased surface expression of AMPA receptors was observed in animal models of MS as well as in MS patients [127]. Of note, the significantly increased levels of TNFα/IL1β in MS/EAE, lead to an increased surface expression of the Ca^2+^-permeable AMPA receptors that lack the GluA2 subunit and thus lead to an enhancement of excitatory synaptic signaling [85,129,142,162,163,173,292,293,294,392,393]. As mentioned above, the Ca^2+^-permeability of glutamate-gated receptors is of particular importance for excitotoxic effects. High concentrations of TNFα increase not only synaptic but also non-synaptic AMPA receptor expression that further contributes to inflammation-induced glutamate excitotoxicity [127,278,373,392,394]. NMDA glutamate receptors could also be affected [138,139,395,396,397,398,399,400,401,402,403]. Increased surface expression of synaptic or extrasynaptic NMDA receptors in response to TNFα [397,404,405] aggravates glutamate excitotoxicity. This could occur either via Ca^2+^ overload of the postsynaptic compartment (Figure 3) or the formation of pathological glutamate receptor complexes [389] that lead to neurodegeneration and neuronal cell death [386,387,388].
(3) Inflammatory cytokines (IL1β, TNFα) induce decreased surface expression of GABA receptors resulting in an imbalance between excitatory and inhibitory signaling [292,406,407,408,409]. TNFα promotes endocytosis of inhibitory GABA receptors thus leading to a decrease in GABA receptor surface expression [292,408]. In the EAE model of multiple sclerosis, inhibitory GABAergic signaling is diminished [84,85,87,128,129,130,407,408,409]. The TNFα effects on AMPA and GABA receptor trafficking are mediated by neuronal TNFR1 receptors [177,292]. IL1β is also involved in the downregulation of synaptic GABA receptors [128,129,407,408,410,411,412]. On the other hand, IL-1β enhances the surface expression of GABA transporters (GATs), thus promoting increased GABA clearance from the synaptic cleft [413,414,415]. In MS patients, GABA levels are significantly reduced and correlated with increasing physical disability in progressive multiple sclerosis [416].
(4) Elevated levels of TNFα increase glutaminase activity in microglia and induce significant release of glutamate from microglia [108,417,418,419]. Activated astrocytes and invading T cells also contribute to increased levels of glutamate in neuroinflammation [259]. These mechanisms will lead to strong activation of extrasynaptic N-methyl-D-aspartate (NMDA) and non-NMDA glutamate receptors. Activation of extrasynaptic glutamate receptors results in neurotoxic effects and ultimately leads to neuronal cell death via various mechanisms [386,387,388,389]. The underlying mechanisms are still under intense investigation, and likely include dysfunctional Ca^2+^ homeostasis, molecular and structural alterations of the synapse, malfunctional pre- and postsynaptic signaling cascades, mitochondrial dysfunctions, and dysregulation of synapse-dependent transcriptional programs [386,387,388,389].

Collectively, early synapse changes in MS (and mouse models of MS) appear to result from increased levels of inflammatory cytokines. Synapse dysfunctions are likely correlates of the known cognitive disabilities and memory dysfunctions in MS patients [68,71,76,92,279,280,420,421,422,423,424,425,426,427,428,429]. Signals from glial cells, particularly microglia, play a prominent role in synaptic pathology. In support of this suggestion, paralysis of microglia ameliorates EAE [430]. Clearly, the role of microglia in this process is complex. As mentioned, pro-inflammatory microglia (“M1” microglia) can generate pathologically elevated levels of inflammatory cytokines in active MS. M2 microglia can counteract these events and disease pathology. Thus, influencing the differentiation behavior of activated microglia toward the anti-inflammatory M2 microglia subtype will likely provide potential for the development of novel therapeutic strategies in MS. Interestingly, MS susceptibility genes are more frequently associated with microglia functions than with neuronal or astrocyte functions emphasizing the central role of microglia for MS [431,432].

Many of the described synaptic alterations in MS/mouse models of MS can be assigned to the postsynaptic compartment. In glutamatergic ribbon synapses of the retina, also strong alterations of presynaptic events have been observed in EAE [239,240,241]. These alterations included changes in the molecular composition of components of the active zone, presynaptic Ca^2+^ homeostasis, and decreased exocytic and endocytic synaptic vesicle cycling. The detailed underlying mechanisms for these presynaptic changes remain to be elucidated but might possibly also involve glutamatergic excitotoxicity. As mentioned, glutamate excitotoxicity can also affect presynaptic events via presynaptic glutamate receptors [275,276]. Gliotransmitter like IL1β and TNFα could also exert effects on such presynaptic events based on the presence of receptors for IL1β and TNFα at the presynaptic terminal [342]. Disorders of the visual system are frequent symptoms of multiple sclerosis, and the early dysfunctions of retinal ribbon synapses observed in the EAE mouse model of MS [239,240,241] could contribute to these symptoms.

## 9. Conclusions and Outlook

Glial cells play an important role in modulating synaptic activity under normal, healthy conditions. Glial cells are targets and amplifiers of neuroinflammatory signals that if secreted in excessive amounts, damage brain synapses and lead to progressive neurodegeneration and brain dysfunctions. The underlying mechanisms and signaling cascades are not fully understood despite enormous recent scientific advancement. Further analyses will likely provide not only an improved understanding of synaptopathy in MS but will also help in the development of novel therapeutic strategies.

## Data Availability

Not applicable.

## Abbreviations

MS	multiple sclerosis
EAE	experimental autoimmune encephalo-myelitis
CNS	central nervous system
OL	oligodendrocyte
GluT	glutamate transporter
Cav-channel	voltage-gated Ca^2+^-channels
CaMKII	Ca^2+^/calmodulin-dependent protein kinase II
PKA	protein kinase A
CREB	cAMP response element-binding protein
pCREB	phospho-CREB
PSD	postsynaptic density
mGluR	metabotropic glutamate receptor
iGluR	ionotropic glutamate receptor

## References

[1] Walton C., King R., Rechtman L., Kaye W., Leray E., Marrie R.A., Robertson N., La Rocca N., Uitdehaag B., van der Mei I. (2020). Rising prevalence of multiple sclerosis worldwide: Insights from the Atlas of MS, third edition. Mult. Scler. J..

[2] Gbaguidi B., Guillemin F., Soudant M., Debouverie M., Mathey G., Epstein J. (2022). Age-period-cohort analysis of the incidence of multiple sclerosis over twenty years in Lorraine, France. Sci. Rep..

[3] Reich D.S., Lucchinetti C.F., Calabresi P.A. (2018). Multiple Sclerosis. N. Engl. J. Med..

[4] Coyle P.K. (2021). What Can We Learn from Sex Differences in MS?. J. Pers. Med..

[5] Lulu S., Graves J., Waubant E. (2016). Menarche increases relapse risk in pediatric multiple sclerosis. Mult. Scler. J..

[6] Wallin M.T., Culpepper W.J., Campbell J.D., Nelson L.M., Langer-Gould A., Marrie R.A., Cutter G.R., Kaye W.E., Wagner L., Tremlett H. (2019). The prevalence of MS in the United States: A population-based estimate using health claims data. Neurology.

[7] Ramien C., Taenzer A., Lupu A., Heckmann N., Engler J.B., Patas K., Friese M.A., Gold S.M. (2016). Sex effects on inflammatory and neurodegenerative processes in multiple sclerosis. Neurosci. Biobehav. Rev..

[8] Rosati G., Aiello I., Pirastru M.I., Mannu L., Sanna G., Sau G.F., Sotgiu S. (1996). Epidemiology of multiple sclerosis in Northwestern Sardinia: Further evidence for higher frequency in Sardinians compared to other Italians. Neuroepidemiology.

[9] Bufill E., Blesa R., Galan I., Dean G. (1995). Prevalence of multiple sclerosis in the region of Osona, Catalonia, northern Spain. J. Neurol. Neurosurg. Psychiatry.

[10] Compston A. (1990). Risk factors for multiple sclerosis: Race or place?. J. Neurol. Neurosurg. Psychiatry.

[11] International Multiple Sclerosis Genetics (2013). Analysis of immune-related loci identifies 48 new susceptibility variants for multiple sclerosis. Nat. Genet..

[12] Olsson T., Barcellos L.F., Alfredsson L. (2017). Interactions between genetic, lifestyle and environmental risk factors for multiple sclerosis. Nat. Rev. Neurol..

[13] Yuan S., Xiong Y., Larsson S.C. (2021). An atlas on risk factors for multiple sclerosis: A Mendelian randomization study. J. Neurol..

[14] Yamout B.I., Alroughani R. (2018). Multiple Sclerosis. Semin. Neurol..

[15] Dobson R., Giovannoni G. (2019). Multiple sclerosis-a review. Eur. J. Neurol..

[16] Attfield K.E., Jensen L.T., Kaufmann M., Friese M.A., Fugger L. (2022). The immunology of multiple sclerosis. Nat. Rev. Immunol..

[17] Ascherio A., Munger K.L. (2007). Environmental risk factors for multiple sclerosis. Part II: Noninfectious factors. Ann. Neurol..

[18] Thacker E.L., Mirzaei F., Ascherio A. (2006). Infectious mononucleosis and risk for multiple sclerosis: A meta-analysis. Ann. Neurol..

[19] Venkatesan A., Johnson R.T. (2014). Infections and multiple sclerosis. Handb. Clin. Neurol..

[20] Bjornevik K., Cortese M., Healy B.C., Kuhle J., Mina M.J., Leng Y., Elledge S.J., Niebuhr D.W., Scher A.I., Munger K.L. (2022). Longitudinal analysis reveals high prevalence of Epstein-Barr virus associated with multiple sclerosis. Science.

[21] Kaufmann M., Evans H., Schaupp A.L., Engler J.B., Kaur G., Willing A., Kursawe N., Schubert C., Attfield K.E., Fugger L. (2021). Identifying CNS-colonizing T cells as potential therapeutic targets to prevent progression of multiple sclerosis. Med.

[22] Lassmann H., Bradl M. (2017). Multiple sclerosis: Experimental models and reality. Acta Neuropathol..

[23] Mass E., Jacome-Galarza C.E., Blank T., Lazarov T., Durham B.H., Ozkaya N., Pastore A., Schwabenland M., Chung Y.R., Rosenblum M.K. (2017). A somatic mutation in erythro-myeloid progenitors causes neurodegenerative disease. Nature.

[24] Goldmann T., Wieghofer P., Muller P.F., Wolf Y., Varol D., Yona S., Brendecke S.M., Kierdorf K., Staszewski O., Datta M. (2013). A new type of microglia gene targeting shows TAK1 to be pivotal in CNS autoimmune inflammation. Nat. Neurosci..

[25] Ruiz F., Vigne S., Pot C. (2019). Resolution of inflammation during multiple sclerosis. Semin. Immunopathol..

[26] Brambilla R. (2019). The contribution of astrocytes to the neuroinflammatory response in multiple sclerosis and experimental autoimmune encephalomyelitis. Acta Neuropathol..

[27] das Neves S.P., Sousa J.C., Sousa N., Cerqueira J.J., Marques F. (2021). Altered astrocytic function in experimental neuroinflammation and multiple sclerosis. Glia.

[28] Seals M.R., Moran M.M., Leavenworth J.D., Leavenworth J.W. (2022). Contribution of Dysregulated B-Cells and IgE Antibody Responses to Multiple Sclerosis. Front. Immunol..

[29] Mathias A., Perriard G., Canales M., Soneson C., Delorenzi M., Schluep M., Du Pasquier R.A. (2017). Increased ex vivo antigen presentation profile of B cells in multiple sclerosis. Mult. Scler. J..

[30] Bar-Or A., Fawaz L., Fan B., Darlington P.J., Rieger A., Ghorayeb C., Calabresi P.A., Waubant E., Hauser S.L., Zhang J. (2010). Abnormal B-cell cytokine responses a trigger of T-cell-mediated disease in MS?. Ann. Neurol..

[31] Li R., Rezk A., Miyazaki Y., Hilgenberg E., Touil H., Shen P., Moore C.S., Michel L., Althekair F., Rajasekharan S. (2015). Proinflammatory GM-CSF-producing B cells in multiple sclerosis and B cell depletion therapy. Sci. Transl. Med..

[32] Bar-Or A., Calabresi P.A., Arnold D., Markowitz C., Shafer S., Kasper L.H., Waubant E., Gazda S., Fox R.J., Panzara M. (2008). Rituximab in relapsing-remitting multiple sclerosis: A 72-week, open-label, phase I trial. Ann. Neurol..

[33] Hauser S.L., Bar-Or A., Comi G., Giovannoni G., Hartung H.P., Hemmer B., Lublin F., Montalban X., Rammohan K.W., Selmaj K. (2017). Ocrelizumab versus Interferon Beta-1a in Relapsing Multiple Sclerosis. N. Engl. J. Med..

[34] Montalban X., Hauser S.L., Kappos L., Arnold D.L., Bar-Or A., Comi G., de Seze J., Giovannoni G., Hartung H.P., Hemmer B. (2017). Ocrelizumab versus Placebo in Primary Progressive Multiple Sclerosis. N. Engl. J. Med..

[35] Myhr K.M., Torkildsen O., Lossius A., Bo L., Holmoy T. (2019). B cell depletion in the treatment of multiple sclerosis. Expert Opin. Biol. Ther..

[36] Compston A., Coles A. (2008). Multiple sclerosis. Lancet.

[37] Sakai R.E., Feller D.J., Galetta K.M., Galetta S.L., Balcer L.J. (2011). Vision in multiple sclerosis: The story, structure-function correlations, and models for neuroprotection. J. Neuroophthalmol..

[38] Ford H. (2020). Clinical presentation and diagnosis of multiple sclerosis. Clin. Med..

[39] Ghasemi N., Razavi S., Nikzad E. (2017). Multiple Sclerosis: Pathogenesis, Symptoms, Diagnoses and Cell-Based Therapy. Cell J..

[40] Wilkins A. (2017). Cerebellar Dysfunction in Multiple Sclerosis. Front. Neurol..

[41] Mross K., Jankowska M., Meller A., Machowska-Sempruch K., Nowacki P., Masztalewicz M., Pawlukowska W. (2022). Sensory Integration Disorders in Patients with Multiple Sclerosis. J. Clin. Med..

[42] Biname F., Pham-Van L.D., Bagnard D. (2021). Manipulating oligodendrocyte intrinsic regeneration mechanism to promote remyelination. Cell. Mol. Life Sci..

[43] Lassmann H., van Horssen J., Mahad D. (2012). Progressive multiple sclerosis: Pathology and pathogenesis. Nat. Rev. Neurol..

[44] Confavreux C., Vukusic S., Moreau T., Adeleine P. (2000). Relapses and progression of disability in multiple sclerosis. N. Engl. J. Med..

[45] Dendrou C.A., Fugger L., Friese M.A. (2015). Immunopathology of multiple sclerosis. Nat. Rev. Immunol..

[46] Schlager C., Korner H., Krueger M., Vidoli S., Haberl M., Mielke D., Brylla E., Issekutz T., Cabanas C., Nelson P.J. (2016). Effector T-cell trafficking between the leptomeninges and the cerebrospinal fluid. Nature.

[47] Quinn J.L., Kumar G., Agasing A., Ko R.M., Axtell R.C. (2018). Role of TFH Cells in Promoting T Helper 17-Induced Neuroinflammation. Front. Immunol..

[48] Galicia G., Boulianne B., Pikor N., Martin A., Gommerman J.L. (2013). Secondary B cell receptor diversification is necessary for T cell mediated neuro-inflammation during experimental autoimmune encephalomyelitis. PLoS ONE.

[49] Kuerten S., Lanz T.V., Lingampalli N., Lahey L.J., Kleinschnitz C., Maurer M., Schroeter M., Braune S., Ziemssen T., Ho P.P. (2020). Autoantibodies against central nervous system antigens in a subset of B cell-dominant multiple sclerosis patients. Proc. Natl. Acad. Sci. USA.

[50] Kuerten S., Jackson L.J., Kaye J., Vollmer T.L. (2018). Impact of Glatiramer Acetate on B Cell-Mediated Pathogenesis of Multiple Sclerosis. CNS Drugs.

[51] Wanleenuwat P., Iwanowski P. (2019). Role of B cells and antibodies in multiple sclerosis. Mult. Scler. Relat. Disord..

[52] Chunder R., Weier A., Maurer H., Luber N., Enders M., Luber G., Heider T., Spitzer A., Tacke S., Becker-Gotot J. (2022). Antibody cross-reactivity between casein and myelin-associated glycoprotein results in central nervous system demyelination. Proc. Natl. Acad. Sci. USA.

[53] Chunder R., Schropp V., Kuerten S. (2020). B Cells in Multiple Sclerosis and Virus-Induced Neuroinflammation. Front. Neurol..

[54] Tengvall K., Huang J., Hellstrom C., Kammer P., Bistrom M., Ayoglu B., Lima Bomfim I., Stridh P., Butt J., Brenner N. (2019). Molecular mimicry between Anoctamin 2 and Epstein-Barr virus nuclear antigen 1 associates with multiple sclerosis risk. Proc. Natl. Acad. Sci. USA.

[55] Schirmer L., Schafer D.P., Bartels T., Rowitch D.H., Calabresi P.A. (2021). Diversity and Function of Glial Cell Types in Multiple Sclerosis. Trends Immunol..

[56] Sen M.K., Mahns D.A., Coorssen J.R., Shortland P.J. (2022). The roles of microglia and astrocytes in phagocytosis and myelination: Insights from the cuprizone model of multiple sclerosis. Glia.

[57] Healy L.M., Stratton J.A., Kuhlmann T., Antel J. (2022). The role of glial cells in multiple sclerosis disease progression. Nat. Rev. Neurol..

[58] Kornek B., Storch M.K., Weissert R., Wallstroem E., Stefferl A., Olsson T., Linington C., Schmidbauer M., Lassmann H. (2000). Multiple sclerosis and chronic autoimmune encephalomyelitis: A comparative quantitative study of axonal injury in active, inactive, and remyelinated lesions. Am. J. Pathol..

[59] Kutzelnigg A., Lucchinetti C.F., Stadelmann C., Bruck W., Rauschka H., Bergmann M., Schmidbauer M., Parisi J.E., Lassmann H. (2005). Cortical demyelination and diffuse white matter injury in multiple sclerosis. Brain.

[60] DeLuca G.C., Williams K., Evangelou N., Ebers G.C., Esiri M.M. (2006). The contribution of demyelination to axonal loss in multiple sclerosis. Brain.

[61] Nikic I., Merkler D., Sorbara C., Brinkoetter M., Kreutzfeldt M., Bareyre F.M., Bruck W., Bishop D., Misgeld T., Kerschensteiner M. (2011). A reversible form of axon damage in experimental autoimmune encephalomyelitis and multiple sclerosis. Nat. Med..

[62] Dziedzic T., Metz I., Dallenga T., Konig F.B., Muller S., Stadelmann C., Bruck W. (2010). Wallerian degeneration: A major component of early axonal pathology in multiple sclerosis. Brain Pathol..

[63] Salapa H.E., Lee S., Shin Y., Levin M.C. (2017). Contribution of the Degeneration of the Neuro-Axonal Unit to the Pathogenesis of Multiple Sclerosis. Brain Sci..

[64] Carassiti D., Altmann D.R., Petrova N., Pakkenberg B., Scaravilli F., Schmierer K. (2018). Neuronal loss, demyelination and volume change in the multiple sclerosis neocortex. Neuropathol. Appl. Neurobiol..

[65] Luchicchi A., Hart B., Frigerio I., van Dam A.M., Perna L., Offerhaus H.L., Stys P.K., Schenk G.J., Geurts J.J.G. (2021). Axon-Myelin Unit Blistering as Early Event in MS Normal Appearing White Matter. Ann. Neurol..

[66] Friese M.A., Schattling B., Fugger L. (2014). Mechanisms of neurodegeneration and axonal dysfunction in multiple sclerosis. Nat. Rev. Neurol..

[67] Derfuss T., Parikh K., Velhin S., Braun M., Mathey E., Krumbholz M., Kumpfel T., Moldenhauer A., Rader C., Sonderegger P. (2009). Contactin-2/TAG-1-directed autoimmunity is identified in multiple sclerosis patients and mediates gray matter pathology in animals. Proc. Natl. Acad. Sci. USA.

[68] Dutta R., Chang A., Doud M.K., Kidd G.J., Ribaudo M.V., Young E.A., Fox R.J., Staugaitis S.M., Trapp B.D. (2011). Demyelination causes synaptic alterations in hippocampi from multiple sclerosis patients. Ann. Neurol..

[69] Rossi S., Motta C., Studer V., Barbieri F., Buttari F., Bergami A., Sancesario G., Bernardini S., De Angelis G., Martino G. (2014). Tumor necrosis factor is elevated in progressive multiple sclerosis and causes excitotoxic neurodegeneration. Mult. Scler. J..

[70] Mandolesi G., Gentile A., Musella A., Fresegna D., De Vito F., Bullitta S., Sepman H., Marfia G.A., Centonze D. (2015). Synaptopathy connects inflammation and neurodegeneration in multiple sclerosis. Nat. Rev. Neurol..

[71] Calabrese M., Magliozzi R., Ciccarelli O., Geurts J.J., Reynolds R., Martin R. (2015). Exploring the origins of grey matter damage in multiple sclerosis. Nat. Rev. Neurosci..

[72] Louapre C., Perlbarg V., Garcia-Lorenzo D., Urbanski M., Benali H., Assouad R., Galanaud D., Freeman L., Bodini B., Papeix C. (2014). Brain networks disconnection in early multiple sclerosis cognitive deficits: An anatomofunctional study. Hum. Brain Mapp..

[73] Steenwijk M.D., Geurts J.J., Daams M., Tijms B.M., Wink A.M., Balk L.J., Tewarie P.K., Uitdehaag B.M., Barkhof F., Vrenken H. (2016). Cortical atrophy patterns in multiple sclerosis are non-random and clinically relevant. Brain.

[74] Bellingacci L., Mancini A., Gaetani L., Tozzi A., Parnetti L., Di Filippo M. (2021). Synaptic Dysfunction in Multiple Sclerosis: A Red Thread from Inflammation to Network Disconnection. Int. J. Mol. Sci..

[75] Di Filippo M., Mancini A., Bellingacci L., Gaetani L., Mazzocchetti P., Zelante T., La Barbera L., De Luca A., Tantucci M., Tozzi A. (2021). Interleukin-17 affects synaptic plasticity and cognition in an experimental model of multiple sclerosis. Cell Rep..

[76] Feuillet L., Reuter F., Audoin B., Malikova I., Barrau K., Cherif A.A., Pelletier J. (2007). Early cognitive impairment in patients with clinically isolated syndrome suggestive of multiple sclerosis. Mult. Scler. J..

[77] Mandolesi G., Grasselli G., Musumeci G., Centonze D. (2010). Cognitive deficits in experimental autoimmune encephalomyelitis: Neuroinflammation and synaptic degeneration. Neurol. Sci..

[78] Haji N., Mandolesi G., Gentile A., Sacchetti L., Fresegna D., Rossi S., Musella A., Sepman H., Motta C., Studer V. (2012). TNF-α-mediated anxiety in a mouse model of multiple sclerosis. Exp. Neurol..

[79] Tarasiuk J., Kapica-Topczewska K., Czarnowska A., Chorazy M., Kochanowicz J., Kulakowska A. (2021). Co-occurrence of Fatigue and Depression in People with Multiple Sclerosis: A Mini-Review. Front. Neurol..

[80] Yirmiya R., Goshen I. (2011). Immune modulation of learning, memory, neural plasticity and neurogenesis. Brain Behav. Immun..

[81] Acharjee S., Nayani N., Tsutsui M., Hill M.N., Ousman S.S., Pittman Q.J. (2013). Altered cognitive-emotional behavior in early experimental autoimmune encephalitis-cytokine and hormonal correlates. Brain Behav. Immun..

[82] Osso L.A., Chan J.R. (2015). Astrocytes Underlie Neuroinflammatory Memory Impairment. Cell.

[83] Chung W.S., Welsh C.A., Barres B.A., Stevens B. (2015). Do glia drive synaptic and cognitive impairment in disease?. Nat. Neurosci..

[84] Stampanoni Bassi M., Mori F., Buttari F., Marfia G.A., Sancesario A., Centonze D., Iezzi E. (2017). Neurophysiology of synaptic functioning in multiple sclerosis. Clin. Neurophysiol..

[85] Centonze D., Muzio L., Rossi S., Cavasinni F., De Chiara V., Bergami A., Musella A., D’Amelio M., Cavallucci V., Martorana A. (2009). Inflammation triggers synaptic alteration and degeneration in experimental autoimmune encephalomyelitis. J. Neurosci..

[86] Audoin B., Zaaraoui W., Reuter F., Rico A., Malikova I., Confort-Gouny S., Cozzone P.J., Pelletier J., Ranjeva J.P. (2010). Atrophy mainly affects the limbic system and the deep grey matter at the first stage of multiple sclerosis. J. Neurol. Neurosurg. Psychiatry.

[87] Di Filippo M., de Iure A., Durante V., Gaetani L., Mancini A., Sarchielli P., Calabresi P. (2015). Synaptic plasticity and experimental autoimmune encephalomyelitis: Implications for multiple sclerosis. Brain Res..

[88] Shi J., Baxter L.C., Kuniyoshi S.M. (2014). Pathologic and imaging correlates of cognitive deficits in multiple sclerosis: Changing the paradigm of diagnosis and prognosis. Cogn. Behav. Neurol..

[89] DeLuca G.C., Yates R.L., Beale H., Morrow S.A. (2015). Cognitive impairment in multiple sclerosis: Clinical, radiologic and pathologic insights. Brain Pathol..

[90] Eshaghi A., Marinescu R.V., Young A.L., Firth N.C., Prados F., Jorge Cardoso M., Tur C., De Angelis F., Cawley N., Brownlee W.J. (2018). Progression of regional grey matter atrophy in multiple sclerosis. Brain.

[91] Solana E., Martinez-Heras E., Montal V., Vilaplana E., Lopez-Soley E., Radua J., Sola-Valls N., Montejo C., Blanco Y., Pulido-Valdeolivas I. (2021). Regional grey matter microstructural changes and volume loss according to disease duration in multiple sclerosis patients. Sci. Rep..

[92] Jurgens T., Jafari M., Kreutzfeldt M., Bahn E., Bruck W., Kerschensteiner M., Merkler D. (2016). Reconstruction of single cortical projection neurons reveals primary spine loss in multiple sclerosis. Brain.

[93] Friese M.A. (2016). Widespread synaptic loss in multiple sclerosis. Brain.

[94] Gentile A., De Vito F., Fresegna D., Rizzo F.R., Bullitta S., Guadalupi L., Vanni V., Buttari F., Stampanoni Bassi M., Leuti A. (2020). Peripheral T cells from multiple sclerosis patients trigger synaptotoxic alterations in central neurons. Neuropathol. Appl. Neurobiol..

[95] Schattling B., Engler J.B., Volkmann C., Rothammer N., Woo M.S., Petersen M., Winkler I., Kaufmann M., Rosenkranz S.C., Fejtova A. (2019). Bassoon proteinopathy drives neurodegeneration in multiple sclerosis. Nat. Neurosci..

[96] Yang G., Parkhurst C.N., Hayes S., Gan W.B. (2013). Peripheral elevation of TNF-α leads to early synaptic abnormalities in the mouse somatosensory cortex in experimental autoimmune encephalomyelitis. Proc. Natl. Acad. Sci. USA.

[97] Huang L., Lafaille J.J., Yang G. (2021). Learning-dependent dendritic spine plasticity is impaired in spontaneous autoimmune encephalomyelitis. Dev. Neurobiol..

[98] Mendel I., Kerlero de Rosbo N., Ben-Nun A. (1995). A myelin oligodendrocyte glycoprotein peptide induces typical chronic experimental autoimmune encephalomyelitis in H-2b mice: Fine specificity and T cell receptor V beta expression of encephalitogenic T cells. Eur. J. Immunol..

[99] Baxter A.G. (2007). The origin and application of experimental autoimmune encephalomyelitis. Nat. Rev. Immunol..

[100] Procaccini C., De Rosa V., Pucino V., Formisano L., Matarese G. (2015). Animal models of Multiple Sclerosis. Eur. J. Pharmacol..

[101] Constantinescu C.S., Farooqi N., O’Brien K., Gran B. (2011). Experimental autoimmune encephalomyelitis (EAE) as a model for multiple sclerosis (MS). Br. J. Pharmacol..

[102] Mix E., Meyer-Rienecker H., Hartung H.P., Zettl U.K. (2010). Animal models of multiple sclerosis--potentials and limitations. Prog. Neurobiol..

[103] Robinson A.P., Harp C.T., Noronha A., Miller S.D. (2014). The experimental autoimmune encephalomyelitis (EAE) model of MS: Utility for understanding disease pathophysiology and treatment. Handb. Clin. Neurol..

[104] Magliozzi R., Howell O.W., Reeves C., Roncaroli F., Nicholas R., Serafini B., Aloisi F., Reynolds R. (2010). A Gradient of neuronal loss and meningeal inflammation in multiple sclerosis. Ann. Neurol..

[105] Choi S.R., Howell O.W., Carassiti D., Magliozzi R., Gveric D., Muraro P.A., Nicholas R., Roncaroli F., Reynolds R. (2012). Meningeal inflammation plays a role in the pathology of primary progressive multiple sclerosis. Brain.

[106] Rossi S., Furlan R., De Chiara V., Motta C., Studer V., Mori F., Musella A., Bergami A., Muzio L., Bernardi G. (2012). Interleukin-1beta causes synaptic hyperexcitability in multiple sclerosis. Ann. Neurol..

[107] Kempuraj D., Thangavel R., Natteru P.A., Selvakumar G.P., Saeed D., Zahoor H., Zaheer S., Iyer S.S., Zaheer A. (2016). Neuroinflammation Induces Neurodegeneration. J. Neurol. Neurosurg. Spine.

[108] Sood A., Preeti K., Fernandes V., Khatri D.K., Singh S.B. (2021). Glia: A major player in glutamate-GABA dysregulation-mediated neurodegeneration. J. Neurosci. Res..

[109] Sudhof T.C. (2013). Neurotransmitter release: The last millisecond in the life of a synaptic vesicle. Neuron.

[110] Brunger A.T., Leitz J. (2023). The Core Complex of the Ca(2+)-Triggered Presynaptic Fusion Machinery. J. Mol. Biol..

[111] Südhof T.C. (2014). The Molecular Machinery of Neurotransmitter Release (Nobel Lecture). Angew. Chem. Int. Ed..

[112] Sudhof T.C. (2012). The presynaptic active zone. Neuron.

[113] Piao C., Sigrist S.J. (2021). (M)Unc13s in Active Zone Diversity: A Drosophila Perspective. Front. Synaptic Neurosci..

[114] Nanou E., Catterall W.A. (2018). Calcium Channels, Synaptic Plasticity, and Neuropsychiatric Disease. Neuron.

[115] Dolphin A.C., Lee A. (2020). Presynaptic calcium channels: Specialized control of synaptic neurotransmitter release. Nat. Rev. Neurosci..

[116] Moser T., Grabner C.P., Schmitz F. (2020). Sensory Processing at Ribbon Synapses in the Retina and the Cochlea. Physiol. Rev..

[117] Williams B., Maddox J.W., Lee A. (2022). Calcium Channels in Retinal Function and Disease. Annu. Rev. Vis. Sci..

[118] Zhang G., Liu J.B., Yuan H.L., Chen S.Y., Singer J.H., Ke J.B. (2022). Multiple Calcium Channel Types with Unique Expression Patterns Mediate Retinal Signaling at Bipolar Cell Ribbon Synapses. J. Neurosci..

[119] Azarnia Tehran D., Maritzen T. (2022). Endocytic proteins: An expanding repertoire of presynaptic functions. Curr. Opin. Neurobiol..

[120] Bai Y., Wang H., Li C. (2022). SAPAP Scaffold Proteins: From Synaptic Function to Neuropsychiatric Disorders. Cells.

[121] Levy A.M., Gomez-Puertas P., Tumer Z. (2022). Neurodevelopmental Disorders Associated with PSD-95 and Its Interaction Partners. Int. J. Mol. Sci..

[122] Krueger-Burg D., Papadopoulos T., Brose N. (2017). Organizers of inhibitory synapses come of age. Curr. Opin. Neurobiol..

[123] Sudhof T.C. (2018). Towards an Understanding of Synapse Formation. Neuron.

[124] Sudhof T.C. (2017). Synaptic Neurexin Complexes: A Molecular Code for the Logic of Neural Circuits. Cell.

[125] Biederer T., Kaeser P.S., Blanpied T.A. (2017). Transcellular Nanoalignment of Synaptic Function. Neuron.

[126] Sudhof T.C. (2021). The cell biology of synapse formation. J. Cell. Biol..

[127] Newcombe J., Uddin A., Dove R., Patel B., Turski L., Nishizawa Y., Smith T. (2008). Glutamate receptor expression in multiple sclerosis lesions. Brain Pathol..

[128] Mandolesi G., Gentile A., Musella A., Centonze D. (2015). IL-1beta dependent cerebellar synaptopathy in a mouse mode of multiple sclerosis. Cerebellum.

[129] Mandolesi G., Musella A., Gentile A., Grasselli G., Haji N., Sepman H., Fresegna D., Bullitta S., De Vito F., Musumeci G. (2013). Interleukin-1beta alters glutamate transmission at purkinje cell synapses in a mouse model of multiple sclerosis. J. Neurosci..

[130] Mori F., Nistico R., Nicoletti C.G., Zagaglia S., Mandolesi G., Piccinin S., Martino G., Finardi A., Rossini P.M., Marfia G.A. (2016). RANTES correlates with inflammatory activity and synaptic excitability in multiple sclerosis. Mult. Scler. J..

[131] Watkins J.C., Evans R.H. (1981). Excitatory amino acid transmitters. Annu. Rev. Pharmacol. Toxicol..

[132] Zhou Y., Danbolt N.C. (2014). Glutamate as a neurotransmitter in the healthy brain. J. Neural. Transm..

[133] Reiner A., Levitz J. (2018). Glutamatergic Signaling in the Central Nervous System: Ionotropic and Metabotropic Receptors in Concert. Neuron.

[134] Hansen K.B., Wollmuth L.P., Bowie D., Furukawa H., Menniti F.S., Sobolevsky A.I., Swanson G.T., Swanger S.A., Greger I.H., Nakagawa T. (2021). Structure, Function, and Pharmacology of Glutamate Receptor Ion Channels. Pharmacol. Rev..

[135] Stover J.F., Pleines U.E., Morganti-Kossmann M.C., Kossmann T., Lowitzsch K., Kempski O.S. (1997). Neurotransmitters in cerebrospinal fluid reflect pathological activity. Eur. J. Clin. Investig..

[136] Sarchielli P., Greco L., Floridi A., Floridi A., Gallai V. (2003). Excitatory amino acids and multiple sclerosis: Evidence from cerebrospinal fluid. Arch. Neurol..

[137] Srinivasan R., Sailasuta N., Hurd R., Nelson S., Pelletier D. (2005). Evidence of elevated glutamate in multiple sclerosis using magnetic resonance spectroscopy at 3 T. Brain.

[138] Sulkowski G., Dabrowska-Bouta B., Salinska E., Struzynska L. (2014). Modulation of glutamate transport and receptor binding by glutamate receptor antagonists in EAE rat brain. PLoS ONE.

[139] Levite M. (2017). Glutamate, T cells and multiple sclerosis. J. Neural. Transm..

[140] Gentile A., Musella A., De Vito F., Fresegna D., Bullitta S., Rizzo F.R., Centonze D., Mandolesi G. (2018). Laquinimod ameliorates excitotoxic damage by regulating glutamate re-uptake. J. Neuroinflamm..

[141] Traynelis S.F., Wollmuth L.P., McBain C.J., Menniti F.S., Vance K.M., Ogden K.K., Hansen K.B., Yuan H., Myers S.J., Dingledine R. (2010). Glutamate receptor ion channels: Structure, regulation, and function. Pharmacol. Rev..

[142] Beattie M.S., Ferguson A.R., Bresnahan J.C. (2010). AMPA-receptor trafficking and injury-induced cell death. Eur. J. Neurosci..

[143] Greger I.H., Watson J.F., Cull-Candy S.G. (2017). Structural and Functional Architecture of AMPA-Type Glutamate Receptors and Their Auxiliary Proteins. Neuron.

[144] Paoletti P., Bellone C., Zhou Q. (2013). NMDA receptor subunit diversity: Impact on receptor properties, synaptic plasticity and disease. Nat. Rev. Neurosci..

[145] Stroebel D., Casado M., Paoletti P. (2018). Triheteromeric NMDA receptors: From structure to synaptic physiology. Curr. Opin. Physiol..

[146] Miyashita T., Oda Y., Horiuchi J., Yin J.C., Morimoto T., Saitoe M. (2012). Mg^2+^ block of Drosophila NMDA receptors is required for long-term memory formation and CREB-dependent gene expression. Neuron.

[147] Granger A.J., Nicoll R.A. (2014). Expression mechanisms underlying long-term potentiation: A postsynaptic view, 10 years on. Philos. Trans. R. Soc. B.

[148] Malinow R. (2003). AMPA receptor trafficking and long-term potentiation. Philos. Trans. R. Soc. B.

[149] Lisman J., Raghavachari S. (2006). A unified model of the presynaptic and postsynaptic changes during LTP at CA1 synapses. Sci. STKE.

[150] Bliss T.V., Collingridge G.L. (2013). Expression of NMDA receptor-dependent LTP in the hippocampus: Bridging the divide. Mol. Brain.

[151] Luscher C., Malenka R.C. (2012). NMDA receptor-dependent long-term potentiation and long-term depression (LTP/LTD). Cold Spring Harb. Perspect. Biol..

[152] Huganir R.L., Nicoll R.A. (2013). AMPARs and synaptic plasticity: The last 25 years. Neuron.

[153] Chater T.E., Goda Y. (2014). The role of AMPA receptors in postsynaptic mechanisms of synaptic plasticity. Front. Cell. Neurosci..

[154] Herring B.E., Nicoll R.A. (2016). Long-Term Potentiation: From CaMKII to AMPA Receptor Trafficking. Annu. Rev. Physiol..

[155] Lisman J. (2017). Glutamatergic synapses are structurally and biochemically complex because of multiple plasticity processes: Long-term potentiation, long-term depression, short-term potentiation and scaling. Philos. Trans. R. Soc. B.

[156] Yasuda R., Hayashi Y., Hell J.W. (2022). CaMKII: A central molecular organizer of synaptic plasticity, learning and memory. Nat. Rev. Neurosci..

[157] Citri A., Malenka R.C. (2008). Synaptic plasticity: Multiple forms, functions, and mechanisms. Neuropsychopharmacology.

[158] Kessels H.W., Malinow R. (2009). Synaptic AMPA receptor plasticity and behavior. Neuron.

[159] Nabavi S., Fox R., Proulx C.D., Lin J.Y., Tsien R.Y., Malinow R. (2014). Engineering a memory with LTD and LTP. Nature.

[160] Malenka R.C., Bear M.F. (2004). LTP and LTD: An embarrassment of riches. Neuron.

[161] Derkach V.A., Oh M.C., Guire E.S., Soderling T.R. (2007). Regulatory mechanisms of AMPA receptors in synaptic plasticity. Nat. Rev. Neurosci..

[162] Beattie E.C., Stellwagen D., Morishita W., Bresnahan J.C., Ha B.K., Von Zastrow M., Beattie M.S., Malenka R.C. (2002). Control of synaptic strength by glial TNFα. Science.

[163] Stellwagen D., Malenka R.C. (2006). Synaptic scaling mediated by glial TNF-α. Nature.

[164] Kann O., Kovacs R. (2007). Mitochondria and neuronal activity. Am. J. Physiol. Cell Physiol..

[165] Faria-Pereira A., Morais V.A. (2022). Synapses: The Brain’s Energy-Demanding Sites. Int. J. Mol. Sci..

[166] Johnson J.E., Perkins G.A., Giddabasappa A., Chaney S., Xiao W., White A.D., Brown J.M., Waggoner J., Ellisman M.H., Fox D.A. (2007). Spatiotemporal regulation of ATP and Ca^2+^ dynamics in vertebrate rod and cone ribbon synapses. Mol. Vis..

[167] Huang L., Jin J., Chen K., You S., Zhang H., Sideris A., Norcini M., Recio-Pinto E., Wang J., Gan W.B. (2021). BDNF produced by cerebral microglia promotes cortical plasticity and pain hypersensitivity after peripheral nerve injury. PLoS Biol..

[168] Castillo P.E. (2012). Presynaptic LTP and LTD of excitatory and inhibitory synapses. Cold Spring Harb. Perspect. Biol..

[169] Turrigiano G. (2012). Homeostatic synaptic plasticity: Local and global mechanisms for stabilizing neuronal function. Cold Spring Harb. Perspect. Biol..

[170] Turrigiano G.G. (2008). The self-tuning neuron: Synaptic scaling of excitatory synapses. Cell.

[171] Vitureira N., Goda Y. (2013). Cell biology in neuroscience: The interplay between Hebbian and homeostatic synaptic plasticity. J. Cell Biol..

[172] Vitureira N., Letellier M., Goda Y. (2012). Homeostatic synaptic plasticity: From single synapses to neural circuits. Curr. Opin. Neurobiol..

[173] Pribiag H., Stellwagen D. (2014). Neuroimmune regulation of homeostatic synaptic plasticity. Neuropharmacology.

[174] Fernandes D., Carvalho A.L. (2016). Mechanisms of homeostatic plasticity in the excitatory synapse. J. Neurochem..

[175] Herculano-Houzel S. (2009). The human brain in numbers: A linearly scaled-up primate brain. Front. Hum. Neurosci..

[176] Sofroniew M.V., Vinters H.V. (2010). Astrocytes: Biology and pathology. Acta Neuropathol..

[177] Chung W.S., Allen N.J., Eroglu C. (2015). Astrocytes Control Synapse Formation, Function, and Elimination. Cold Spring Harb. Perspect. Biol..

[178] Escartin C., Galea E., Lakatos A., O’Callaghan J.P., Petzold G.C., Serrano-Pozo A., Steinhauser C., Volterra A., Carmignoto G., Agarwal A. (2021). Reactive astrocyte nomenclature, definitions, and future directions. Nat. Neurosci..

[179] Simard M., Nedergaard M. (2004). The neurobiology of glia in the context of water and ion homeostasis. Neuroscience.

[180] Deitmer J.W., Hatton G.I., Parpura V. (2004). pH regulation and acid/base-mediated transport in glial cells. Glial ⇔ Neuronal Signaling.

[181] Brown A.M., Ransom B.R. (2007). Astrocyte glycogen and brain energy metabolism. Glia.

[182] Attwell D., Buchan A.M., Charpak S., Lauritzen M., Macvicar B.A., Newman E.A. (2010). Glial and neuronal control of brain blood flow. Nature.

[183] Nedergaard M. (2013). Garbage truck of the brain. Science.

[184] Verkhratsky A., Nedergaard M. (2016). The homeostatic astroglia emerges from evolutionary specialization of neural cells. Philos. Trans. R. Soc. B.

[185] Zuchero J.B., Barres B.A. (2015). Glia in mammalian development and disease. Development.

[186] Chao T., Rickmann M., Wolff J., Volterra A., Magistretti P., Haydon P. (2002). The synapse-astrocyte boundary: Anatomical basis for an integrative role of glia in synaptic transmission. Tripertite Synapses: Synaptic Transmission with Glia.

[187] Farmer W.T., Murai K. (2017). Resolving Astrocyte Heterogeneity in the CNS. Front. Cell. Neurosci..

[188] Chai H., Diaz-Castro B., Shigetomi E., Monte E., Octeau J.C., Yu X., Cohn W., Rajendran P.S., Vondriska T.M., Whitelegge J.P. (2017). Neural Circuit-Specialized Astrocytes: Transcriptomic, Proteomic, Morphological, and Functional Evidence. Neuron.

[189] Bushong E.A., Martone M.E., Jones Y.Z., Ellisman M.H. (2002). Protoplasmic astrocytes in CA1 stratum radiatum occupy separate anatomical domains. J. Neurosci..

[190] Halassa M.M., Fellin T., Haydon P.G. (2007). The tripartite synapse: Roles for gliotransmission in health and disease. Trends Mol. Med..

[191] Oberheim N.A., Takano T., Han X., He W., Lin J.H., Wang F., Xu Q., Wyatt J.D., Pilcher W., Ojemann J.G. (2009). Uniquely hominid features of adult human astrocytes. J. Neurosci..

[192] Ventura R., Harris K.M. (1999). Three-dimensional relationships between hippocampal synapses and astrocytes. J. Neurosci..

[193] Perea G., Araque A. (2007). Astrocytes potentiate transmitter release at single hippocampal synapses. Science.

[194] Verkhratsky A., Nedergaard M. (2014). Astroglial cradle in the life of the synapse. Philos. Trans. R. Soc. B.

[195] Bezzi P., Volterra A. (2001). A neuron-glia signalling network in the active brain. Curr. Opin. Neurobiol..

[196] Panatier A., Robitaille R. (2016). Astrocytic mGluR5 and the tripartite synapse. Neuroscience.

[197] De Pitta M., Brunel N., Volterra A. (2016). Astrocytes: Orchestrating synaptic plasticity?. Neuroscience.

[198] Eroglu C., Barres B.A. (2010). Regulation of synaptic connectivity by glia. Nature.

[199] Colon-Ramos D.A., Margeta M.A., Shen K. (2007). Glia promote local synaptogenesis through UNC-6 (netrin) signaling in *C. elegans*. Science.

[200] Eroglu C., Allen N.J., Susman M.W., O’Rourke N.A., Park C.Y., Ozkan E., Chakraborty C., Mulinyawe S.B., Annis D.S., Huberman A.D. (2009). Gabapentin receptor α2delta-1 is a neuronal thrombospondin receptor responsible for excitatory CNS synaptogenesis. Cell.

[201] Crawford D.C., Jiang X., Taylor A., Mennerick S. (2012). Astrocyte-derived thrombospondins mediate the development of hippocampal presynaptic plasticity in vitro. J. Neurosci..

[202] Fuentes-Medel Y., Ashley J., Barria R., Maloney R., Freeman M., Budnik V. (2012). Integration of a retrograde signal during synapse formation by glia-secreted TGF-beta ligand. Curr. Biol..

[203] Risher W.C., Eroglu C. (2012). Thrombospondins as key regulators of synaptogenesis in the central nervous system. Matrix Biol..

[204] Singh S.K., Stogsdill J.A., Pulimood N.S., Dingsdale H., Kim Y.H., Pilaz L.J., Kim I.H., Manhaes A.C., Rodrigues W.S., Pamukcu A. (2016). Astrocytes Assemble Thalamocortical Synapses by Bridging NRX1α and NL1 via Hevin. Cell.

[205] Tong X.J., Lopez-Soto E.J., Li L., Liu H., Nedelcu D., Lipscombe D., Hu Z., Kaplan J.M. (2017). Retrograde Synaptic Inhibition Is Mediated by α-Neurexin Binding to the α2δ Subunits of N-Type Calcium Channels. Neuron.

[206] Song I., Dityatev A. (2018). Crosstalk between glia, extracellular matrix and neurons. Brain Res. Bull..

[207] Farhy-Tselnicker I., van Casteren A.C.M., Lee A., Chang V.T., Aricescu A.R., Allen N.J. (2017). Astrocyte-Secreted Glypican 4 Regulates Release of Neuronal Pentraxin 1 from Axons to Induce Functional Synapse Formation. Neuron.

[208] Liddelow S.A., Barres B.A. (2017). Reactive Astrocytes: Production, Function, and Therapeutic Potential. Immunity.

[209] Allen N.J. (2014). Astrocyte regulation of synaptic behavior. Annu. Rev. Cell Dev. Biol..

[210] Allen N.J., Bennett M.L., Foo L.C., Wang G.X., Chakraborty C., Smith S.J., Barres B.A. (2012). Astrocyte glypicans 4 and 6 promote formation of excitatory synapses via GluA1 AMPA receptors. Nature.

[211] Anderson C.M., Swanson R.A. (2000). Astrocyte glutamate transport: Review of properties, regulation, and physiological functions. Glia.

[212] Oliet S.H., Piet R., Poulain D.A. (2001). Control of glutamate clearance and synaptic efficacy by glial coverage of neurons. Science.

[213] Conti F., Minelli A., Melone M. (2004). GABA transporters in the mammalian cerebral cortex: Localization, development and pathological implications. Brain Res. Rev..

[214] Beenhakker M.P., Huguenard J.R. (2010). Astrocytes as gatekeepers of GABAB receptor function. J. Neurosci..

[215] Tanaka M., Shih P.Y., Gomi H., Yoshida T., Nakai J., Ando R., Furuichi T., Mikoshiba K., Semyanov A., Itohara S. (2013). Astrocytic Ca^2+^ signals are required for the functional integrity of tripartite synapses. Mol. Brain.

[216] Kettenmann H., Kirchhoff F., Verkhratsky A. (2013). Microglia: New roles for the synaptic stripper. Neuron.

[217] Khakh B.S., Sofroniew M.V. (2015). Diversity of astrocyte functions and phenotypes in neural circuits. Nat. Neurosci..

[218] Liddelow S., Barres B. (2015). SnapShot: Astrocytes in Health and Disease. Cell.

[219] Lopez-Colome A.M., Lopez E., Mendez-Flores O.G., Ortega A. (2016). Glutamate Receptor Stimulation Up-Regulates Glutamate Uptake in Human Muller Glia Cells. Neurochem. Res..

[220] Danbolt N.C., Furness D.N., Zhou Y. (2016). Neuronal vs. glial glutamate uptake: Resolving the conundrum. Neurochem. Int..

[221] Grewer C., Gameiro A., Rauen T. (2014). SLC1 glutamate transporters. Pflügers Arch..

[222] Danbolt N.C. (2001). Glutamate uptake. Prog. Neurobiol..

[223] Pines G., Danbolt N.C., Bjoras M., Zhang Y., Bendahan A., Eide L., Koepsell H., Storm-Mathisen J., Seeberg E., Kanner B.I. (1992). Cloning and expression of a rat brain L-glutamate transporter. Nature.

[224] Storck T., Schulte S., Hofmann K., Stoffel W. (1992). Structure, expression, and functional analysis of a Na(+)-dependent glutamate/aspartate transporter from rat brain. Proc. Natl. Acad. Sci. USA.

[225] Kanai Y., Hediger M.A. (1992). Primary structure and functional characterization of a high-affinity glutamate transporter. Nature.

[226] Fairman W.A., Vandenberg R.J., Arriza J.L., Kavanaugh M.P., Amara S.G. (1995). An excitatory amino-acid transporter with properties of a ligand-gated chloride channel. Nature.

[227] Palmer M.J., Taschenberger H., Hull C., Tremere L., von Gersdorff H. (2003). Synaptic activation of presynaptic glutamate transporter currents in nerve terminals. J. Neurosci..

[228] Hasegawa J., Obara T., Tanaka K., Tachibana M. (2006). High-density presynaptic transporters are required for glutamate removal from the first visual synapse. Neuron.

[229] Furness D.N., Dehnes Y., Akhtar A.Q., Rossi D.J., Hamann M., Grutle N.J., Gundersen V., Holmseth S., Lehre K.P., Ullensvang K. (2008). A quantitative assessment of glutamate uptake into hippocampal synaptic terminals and astrocytes: New insights into a neuronal role for excitatory amino acid transporter 2 (EAAT2). Neuroscience.

[230] Reichenbach A., Bringmann A. (2013). New functions of Muller cells. Glia.

[231] Rimmele T.S., Rosenberg P.A. (2016). GLT-1: The elusive presynaptic glutamate transporter. Neurochem. Int..

[232] Murphy-Royal C., Dupuis J., Groc L., Oliet S.H.R. (2017). Astroglial glutamate transporters in the brain: Regulating neurotransmitter homeostasis and synaptic transmission. J. Neurosci. Res..

[233] Halassa M.M., Fellin T., Takano H., Dong J.H., Haydon P.G. (2007). Synaptic islands defined by the territory of a single astrocyte. J. Neurosci..

[234] Tanaka K., Watase K., Manabe T., Yamada K., Watanabe M., Takahashi K., Iwama H., Nishikawa T., Ichihara N., Kikuchi T. (1997). Epilepsy and exacerbation of brain injury in mice lacking the glutamate transporter GLT-1. Science.

[235] Schmitt A., Asan E., Lesch K.P., Kugler P. (2002). A splice variant of glutamate transporter GLT1/EAAT2 expressed in neurons: Cloning and localization in rat nervous system. Neuroscience.

[236] Reye P., Sullivan R., Fletcher E.L., Pow D.V. (2002). Distribution of two splice variants of the glutamate transporter GLT1 in the retinas of humans, monkeys, rabbits, rats, cats, and chickens. J. Comp. Neurol..

[237] Kugler P., Schmitt A. (2003). Complementary neuronal and glial expression of two high-affinity glutamate transporter GLT1/EAAT2 forms in rat cerebral cortex. Histochem. Cell Biol..

[238] Tang F.S., Yuan H.L., Liu J.B., Zhang G., Chen S.Y., Ke J.B. (2022). Glutamate Transporters EAAT2 and EAAT5 Differentially Shape Synaptic Transmission from Rod Bipolar Cell Terminals. eNeuro.

[239] Dembla M., Kesharwani A., Natarajan S., Fecher-Trost C., Fairless R., Williams S.K., Flockerzi V., Diem R., Schwarz K., Schmitz F. (2018). Early auto-immune targeting of photoreceptor ribbon synapses in mouse models of multiple sclerosis. EMBO Mol. Med..

[240] Mukherjee A., Katiyar R., Dembla E., Dembla M., Kumar P., Belkacemi A., Jung M., Beck A., Flockerzi V., Schwarz K. (2020). Disturbed Presynaptic Ca^2+^ Signaling in Photoreceptors in the EAE Mouse Model of Multiple Sclerosis. iScience.

[241] Kesharwani A., Schwarz K., Dembla E., Dembla M., Schmitz F. (2021). Early Changes in Exo- and Endocytosis in the EAE Mouse Model of Multiple Sclerosis Correlate with Decreased Synaptic Ribbon Size and Reduced Ribbon-Associated Vesicle Pools in Rod Photoreceptor Synapses. Int. J. Mol. Sci..

[242] Bennett J.L., Costello F., Chen J.J., Petzold A., Biousse V., Newman N.J., Galetta S.L. (2023). Optic neuritis and autoimmune optic neuropathies: Advances in diagnosis and treatment. Lancet Neurol..

[243] Lehre K.P., Davanger S., Danbolt N.C. (1997). Localization of the glutamate transporter protein GLAST in rat retina. Brain Res..

[244] Derouiche A., Rauen T. (1995). Coincidence of L-glutamate/L-aspartate transporter (GLAST) and glutamine synthetase (GS) immunoreactions in retinal glia: Evidence for coupling of GLAST and GS in transmitter clearance. J. Neurosci. Res..

[245] Rauen T., Rothstein J.D., Wassle H. (1996). Differential expression of three glutamate transporter subtypes in the rat retina. Cell Tissue Res..

[246] Rauen T., Taylor W.R., Kuhlbrodt K., Wiessner M. (1998). High-affinity glutamate transporters in the rat retina: A major role of the glial glutamate transporter GLAST-1 in transmitter clearance. Cell Tissue Res..

[247] Harada T., Harada C., Watanabe M., Inoue Y., Sakagawa T., Nakayama N., Sasaki S., Okuyama S., Watase K., Wada K. (1998). Functions of the two glutamate transporters GLAST and GLT-1 in the retina. Proc. Natl. Acad. Sci. USA.

[248] Pow D.V., Barnett N.L. (1999). Changing patterns of spatial buffering of glutamate in developing rat retinae are mediated by the Muller cell glutamate transporter GLAST. Cell Tissue Res..

[249] Kugler P., Beyer A. (2003). Expression of glutamate transporters in human and rat retina and rat optic nerve. Histochem. Cell Biol..

[250] Fyk-Kolodziej B., Qin P., Dzhagaryan A., Pourcho R.G. (2004). Differential cellular and subcellular distribution of glutamate transporters in the cat retina. Vis. Neurosci..

[251] Sarthy V.P., Pignataro L., Pannicke T., Weick M., Reichenbach A., Harada T., Tanaka K., Marc R. (2005). Glutamate transport by retinal Muller cells in glutamate/aspartate transporter-knockout mice. Glia.

[252] Broer S., Brookes N. (2001). Transfer of glutamine between astrocytes and neurons. J. Neurochem..

[253] Wersinger E., Schwab Y., Sahel J.A., Rendon A., Pow D.V., Picaud S., Roux M.J. (2006). The glutamate transporter EAAT5 works as a presynaptic receptor in mouse rod bipolar cells. J. Physiol..

[254] Veruki M.L., Morkve S.H., Hartveit E. (2006). Activation of a presynaptic glutamate transporter regulates synaptic transmission through electrical signaling. Nat. Neurosci..

[255] Gehlen J., Aretzweiler C., Mataruga A., Fahlke C., Muller F. (2021). Excitatory Amino Acid Transporter EAAT5 Improves Temporal Resolution in the Retina. eNeuro.

[256] Barbour B., Brew H., Attwell D. (1988). Electrogenic glutamate uptake in glial cells is activated by intracellular potassium. Nature.

[257] Rossi D.J., Oshima T., Attwell D. (2000). Glutamate release in severe brain ischaemia is mainly by reversed uptake. Nature.

[258] Grewer C., Gameiro A., Zhang Z., Tao Z., Braams S., Rauen T. (2008). Glutamate forward and reverse transport: From molecular mechanism to transporter-mediated release after ischemia. IUBMB Life.

[259] Malarkey E.B., Parpura V. (2008). Mechanisms of glutamate release from astrocytes. Neurochem. Int..

[260] Jackman N.A., Uliasz T.F., Hewett J.A., Hewett S.J. (2010). Regulation of system x(c)(-)activity and expression in astrocytes by interleukin-1beta: Implications for hypoxic neuronal injury. Glia.

[261] Lewerenz J., Hewett S.J., Huang Y., Lambros M., Gout P.W., Kalivas P.W., Massie A., Smolders I., Methner A., Pergande M. (2013). The cystine/glutamate antiporter system x(c)(-) in health and disease: From molecular mechanisms to novel therapeutic opportunities. Antioxid. Redox Signal..

[262] Park H., Oh S.J., Han K.S., Woo D.H., Park H., Mannaioni G., Traynelis S.F., Lee C.J. (2009). Bestrophin-1 encodes for the Ca^2+^-activated anion channel in hippocampal astrocytes. J. Neurosci..

[263] Park H., Han K.S., Seo J., Lee J., Dravid S.M., Woo J., Chun H., Cho S., Bae J.Y., An H. (2015). Channel-mediated astrocytic glutamate modulates hippocampal synaptic plasticity by activating postsynaptic NMDA receptors. Mol. Brain.

[264] Woo D.H., Han K.S., Shim J.W., Yoon B.E., Kim E., Bae J.Y., Oh S.J., Hwang E.M., Marmorstein A.D., Bae Y.C. (2012). TREK-1 and Best1 channels mediate fast and slow glutamate release in astrocytes upon GPCR activation. Cell.

[265] Satarker S., Bojja S.L., Gurram P.C., Mudgal J., Arora D., Nampoothiri M. (2022). Astrocytic Glutamatergic Transmission and Its Implications in Neurodegenerative Disorders. Cells.

[266] Junankar P.R., Kirk K. (2000). Organic osmolyte channels: A comparative view. Cell. Physiol. Biochem..

[267] Eggermont J., Trouet D., Carton I., Nilius B. (2001). Cellular function and control of volume-regulated anion channels. Cell. Biochem. Biophys..

[268] Beppu K., Kubo N., Matsui K. (2021). Glial amplification of synaptic signals. J. Physiol..

[269] Jourdain P., Bergersen L.H., Bhaukaurally K., Bezzi P., Santello M., Domercq M., Matute C., Tonello F., Gundersen V., Volterra A. (2007). Glutamate exocytosis from astrocytes controls synaptic strength. Nat. Neurosci..

[270] Halassa M.M., Haydon P.G. (2010). Integrated brain circuits: Astrocytic networks modulate neuronal activity and behavior. Annu. Rev. Physiol..

[271] Sun W., McConnell E., Pare J.F., Xu Q., Chen M., Peng W., Lovatt D., Han X., Smith Y., Nedergaard M. (2013). Glutamate-dependent neuroglial calcium signaling differs between young and adult brain. Science.

[272] Araque A., Carmignoto G., Haydon P.G., Oliet S.H., Robitaille R., Volterra A. (2014). Gliotransmitters travel in time and space. Neuron.

[273] Ceprian M., Fulton D. (2019). Glial Cell AMPA Receptors in Nervous System Health, Injury and Disease. Int. J. Mol. Sci..

[274] Durkee C.A., Araque A. (2019). Diversity and Specificity of Astrocyte-neuron Communication. Neuroscience.

[275] Bezzi P., Domercq M., Brambilla L., Galli R., Schols D., De Clercq E., Vescovi A., Bagetta G., Kollias G., Meldolesi J. (2001). CXCR4-activated astrocyte glutamate release via TNFα: Amplification by microglia triggers neurotoxicity. Nat. Neurosci..

[276] Pascual O., Ben Achour S., Rostaing P., Triller A., Bessis A. (2012). Microglia activation triggers astrocyte-mediated modulation of excitatory neurotransmission. Proc. Natl. Acad. Sci. USA.

[277] Habbas S., Santello M., Becker D., Stubbe H., Zappia G., Liaudet N., Klaus F.R., Kollias G., Fontana A., Pryce C.R. (2015). Neuroinflammatory TNFα Impairs Memory via Astrocyte Signaling. Cell.

[278] Santello M., Volterra A. (2012). TNFα in synaptic function: Switching gears. Trends Neurosci..

[279] Damasceno A., Damasceno B.P., Cendes F. (2014). The clinical impact of cerebellar grey matter pathology in multiple sclerosis. PLoS ONE.

[280] Planche V., Ruet A., Coupe P., Lamargue-Hamel D., Deloire M., Pereira B., Manjon J.V., Munsch F., Moscufo N., Meier D.S. (2017). Hippocampal microstructural damage correlates with memory impairment in clinically isolated syndrome suggestive of multiple sclerosis. Mult. Scler. J..

[281] Newman E.A. (2003). New roles for astrocytes: Regulation of synaptic transmission. Trends Neurosci..

[282] Hamilton N.B., Attwell D. (2010). Do astrocytes really exocytose neurotransmitters?. Nat. Rev. Neurosci..

[283] Burda J.E., Sofroniew M.V. (2017). Seducing astrocytes to the dark side. Cell Res..

[284] Liddelow S.A., Guttenplan K.A., Clarke L.E., Bennett F.C., Bohlen C.J., Schirmer L., Bennett M.L., Munch A.E., Chung W.S., Peterson T.C. (2017). Neurotoxic reactive astrocytes are induced by activated microglia. Nature.

[285] Salter M.W., Stevens B. (2017). Microglia emerge as central players in brain disease. Nat. Med..

[286] Guidotti G., Scarlata C., Brambilla L., Rossi D. (2021). Tumor Necrosis Factor Alpha in Amyotrophic Lateral Sclerosis: Friend or Foe?. Cells.

[287] Fiacco T.A., McCarthy K.D. (2018). Multiple Lines of Evidence Indicate that Gliotransmission does not Occur under Physiological Conditions. J. Neurosci..

[288] Savtchouk I., Volterra A. (2018). Gliotransmission: Beyond Black-and-White. J. Neurosci..

[289] Schwarz Y., Zhao N., Kirchhoff F., Bruns D. (2017). Astrocytes control synaptic strength by two distinct v-SNARE-dependent release pathways. Nat. Neurosci..

[290] Santello M., Bezzi P., Volterra A. (2011). TNFα controls glutamatergic gliotransmission in the hippocampal dentate gyrus. Neuron.

[291] Petrelli F., Bezzi P. (2016). Novel insights into gliotransmitters. Curr. Opin. Pharmacol..

[292] Stellwagen D., Beattie E.C., Seo J.Y., Malenka R.C. (2005). Differential regulation of AMPA receptor and GABA receptor trafficking by tumor necrosis factor-α. J. Neurosci..

[293] Heir R., Stellwagen D. (2020). TNF-Mediated Homeostatic Synaptic Plasticity: From in vitro to in vivo Models. Front. Cell. Neurosci..

[294] Galanis C., Vlachos A. (2020). Hebbian and Homeostatic Synaptic Plasticity-Do Alterations of One Reflect Enhancement of the Other?. Front. Cell. Neurosci..

[295] Vesce S., Rossi D., Brambilla L., Volterra A. (2007). Glutamate release from astrocytes in physiological conditions and in neurodegenerative disorders characterized by neuroinflammation. Int. Rev. Neurobiol..

[296] Duman R.S. (2014). Pathophysiology of depression and innovative treatments: Remodeling glutamatergic synaptic connections. Dialogues Clin. Neurosci..

[297] McEwen B.S., Nasca C., Gray J.D. (2016). Stress Effects on Neuronal Structure: Hippocampus, Amygdala, and Prefrontal Cortex. Neuropsychopharmacology.

[298] Bocchio M., Lukacs I.P., Stacey R., Plaha P., Apostolopoulos V., Livermore L., Sen A., Ansorge O., Gillies M.J., Somogyi P. (2018). Group II Metabotropic Glutamate Receptors Mediate Presynaptic Inhibition of Excitatory Transmission in Pyramidal Neurons of the Human Cerebral Cortex. Front. Cell. Neurosci..

[299] del Rey A., Balschun D., Wetzel W., Randolf A., Besedovsky H.O. (2013). A cytokine network involving brain-borne IL-1β, IL-1ra, IL-18, IL-6, and TNFα operates during long-term potentiation and learning. Brain Behav. Immun..

[300] Kabba J.A., Xu Y., Christian H., Ruan W., Chenai K., Xiang Y., Zhang L., Saavedra J.M., Pang T. (2018). Microglia: Housekeeper of the Central Nervous System. Cell. Mol. Neurobiol..

[301] Hyvarinen T., Hagman S., Ristola M., Sukki L., Veijula K., Kreutzer J., Kallio P., Narkilahti S. (2019). Co-stimulation with IL-1beta and TNF-α induces an inflammatory reactive astrocyte phenotype with neurosupportive characteristics in a human pluripotent stem cell model system. Sci. Rep..

[302] Spurgat M.S., Tang S.J. (2022). Single-Cell RNA-Sequencing: Astrocyte and Microglial Heterogeneity in Health and Disease. Cells.

[303] Lian H., Yang L., Cole A., Sun L., Chiang A.C., Fowler S.W., Shim D.J., Rodriguez-Rivera J., Taglialatela G., Jankowsky J.L. (2015). NFkappaB-activated astroglial release of complement C3 compromises neuronal morphology and function associated with Alzheimer’s disease. Neuron.

[304] Reid J.K., Kuipers H.F. (2021). She Doesn’t Even Go Here: The Role of Inflammatory Astrocytes in CNS Disorders. Front. Cell. Neurosci..

[305] Ingram G., Hakobyan S., Robertson N.P., Morgan B.P. (2009). Complement in multiple sclerosis: Its role in disease and potential as a biomarker. Clin. Exp. Immunol..

[306] Michailidou I., Naessens D.M., Hametner S., Guldenaar W., Kooi E.J., Geurts J.J., Baas F., Lassmann H., Ramaglia V. (2017). Complement C3 on microglial clusters in multiple sclerosis occur in chronic but not acute disease: Implication for disease pathogenesis. Glia.

[307] Michailidou I., Willems J.G., Kooi E.J., van Eden C., Gold S.M., Geurts J.J., Baas F., Huitinga I., Ramaglia V. (2015). Complement C1q-C3-associated synaptic changes in multiple sclerosis hippocampus. Ann. Neurol..

[308] Watkins L.M., Neal J.W., Loveless S., Michailidou I., Ramaglia V., Rees M.I., Reynolds R., Robertson N.P., Morgan B.P., Howell O.W. (2016). Complement is activated in progressive multiple sclerosis cortical grey matter lesions. J. Neuroinflamm..

[309] Stevens B., Allen N.J., Vazquez L.E., Howell G.R., Christopherson K.S., Nouri N., Micheva K.D., Mehalow A.K., Huberman A.D., Stafford B. (2007). The classical complement cascade mediates CNS synapse elimination. Cell.

[310] Stephan A.H., Barres B.A., Stevens B. (2012). The complement system: An unexpected role in synaptic pruning during development and disease. Annu. Rev. Neurosci..

[311] Gomez-Nicola D., Perry V.H. (2015). Microglial dynamics and role in the healthy and diseased brain: A paradigm of functional plasticity. Neuroscientist.

[312] Mittelbronn M., Dietz K., Schluesener H.J., Meyermann R. (2001). Local distribution of microglia in the normal adult human central nervous system differs by up to one order of magnitude. Acta Neuropathol..

[313] Van Hove H., Martens L., Scheyltjens I., De Vlaminck K., Pombo Antunes A.R., De Prijck S., Vandamme N., De Schepper S., Van Isterdael G., Scott C.L. (2019). A single-cell atlas of mouse brain macrophages reveals unique transcriptional identities shaped by ontogeny and tissue environment. Nat. Neurosci..

[314] Jordao M.J.C., Sankowski R., Brendecke S.M., Sagar G., Locatelli G., Tai Y.H., Tay T.L., Schramm E., Armbruster S., Hagemeyer N. (2019). Single-cell profiling identifies myeloid cell subsets with distinct fates during neuroinflammation. Science.

[315] Mrdjen D., Pavlovic A., Hartmann F.J., Schreiner B., Utz S.G., Leung B.P., Lelios I., Heppner F.L., Kipnis J., Merkler D. (2018). High-Dimensional Single-Cell Mapping of Central Nervous System Immune Cells Reveals Distinct Myeloid Subsets in Health, Aging, and Disease. Immunity.

[316] Ginhoux F., Lim S., Hoeffel G., Low D., Huber T. (2013). Origin and differentiation of microglia. Front. Cell. Neurosci..

[317] Masuda T., Amann L., Monaco G., Sankowski R., Staszewski O., Krueger M., Del Gaudio F., He L., Paterson N., Nent E. (2022). Specification of CNS macrophage subsets occurs postnatally in defined niches. Nature.

[318] Aguzzi A., Barres B.A., Bennett M.L. (2013). Microglia: Scapegoat, saboteur, or something else?. Science.

[319] Wolf S.A., Boddeke H.W., Kettenmann H. (2017). Microglia in Physiology and Disease. Annu. Rev. Physiol..

[320] Bennett M.L., Bennett F.C., Liddelow S.A., Ajami B., Zamanian J.L., Fernhoff N.B., Mulinyawe S.B., Bohlen C.J., Adil A., Tucker A. (2016). New tools for studying microglia in the mouse and human CNS. Proc. Natl. Acad. Sci. USA.

[321] Zrzavy T., Hametner S., Wimmer I., Butovsky O., Weiner H.L., Lassmann H. (2017). Loss of ‘homeostatic’ microglia and patterns of their activation in active multiple sclerosis. Brain.

[322] Nimmerjahn A., Kirchhoff F., Helmchen F. (2005). Resting microglial cells are highly dynamic surveillants of brain parenchyma in vivo. Science.

[323] Hanisch U.K., Kettenmann H. (2007). Microglia: Active sensor and versatile effector cells in the normal and pathologic brain. Nat. Neurosci..

[324] Bilimoria P.M., Stevens B. (2015). Microglia function during brain development: New insights from animal models. Brain Res..

[325] Vidal-Itriago A., Radford R.A.W., Aramideh J.A., Maurel C., Scherer N.M., Don E.K., Lee A., Chung R.S., Graeber M.B., Morsch M. (2022). Microglia morphophysiological diversity and its implications for the CNS. Front. Immunol..

[326] Davalos D., Grutzendler J., Yang G., Kim J.V., Zuo Y., Jung S., Littman D.R., Dustin M.L., Gan W.B. (2005). ATP mediates rapid microglial response to local brain injury in vivo. Nat. Neurosci..

[327] Wake H., Moorhouse A.J., Jinno S., Kohsaka S., Nabekura J. (2009). Resting microglia directly monitor the functional state of synapses in vivo and determine the fate of ischemic terminals. J. Neurosci..

[328] Tremblay M.E., Lowery R.L., Majewska A.K. (2010). Microglial interactions with synapses are modulated by visual experience. PLoS Biol..

[329] Schafer D.P., Lehrman E.K., Kautzman A.G., Koyama R., Mardinly A.R., Yamasaki R., Ransohoff R.M., Greenberg M.E., Barres B.A., Stevens B. (2012). Microglia sculpt postnatal neural circuits in an activity and complement-dependent manner. Neuron.

[330] Wang X., Zhao L., Zhang J., Fariss R.N., Ma W., Kretschmer F., Wang M., Qian H.H., Badea T.C., Diamond J.S. (2016). Requirement for Microglia for the Maintenance of Synaptic Function and Integrity in the Mature Retina. J. Neurosci..

[331] Hong S., Stevens B. (2016). Microglia: Phagocytosing to Clear, Sculpt, and Eliminate. Dev. Cell.

[332] Ohgomori T., Iinuma K., Yamada J., Jinno S. (2021). A unique subtype of ramified microglia associated with synapses in the rat hippocampus. Eur. J. Neurosci..

[333] Schafer D.P., Lehrman E.K., Stevens B. (2013). The “quad-partite” synapse: Microglia-synapse interactions in the developing and mature CNS. Glia.

[334] Hristovska I., Pascual O. (2015). Deciphering Resting Microglial Morphology and Process Motility from a Synaptic Prospect. Front. Integr. Neurosci..

[335] Akiyoshi R., Wake H., Kato D., Horiuchi H., Ono R., Ikegami A., Haruwaka K., Omori T., Tachibana Y., Moorhouse A.J. (2018). Microglia Enhance Synapse Activity to Promote Local Network Synchronization. eNeuro.

[336] Fontainhas A.M., Wang M., Liang K.J., Chen S., Mettu P., Damani M., Fariss R.N., Li W., Wong W.T. (2011). Microglial morphology and dynamic behavior is regulated by ionotropic glutamatergic and GABAergic neurotransmission. PLoS ONE.

[337] Dissing-Olesen L., LeDue J.M., Rungta R.L., Hefendehl J.K., Choi H.B., MacVicar B.A. (2014). Activation of neuronal NMDA receptors triggers transient ATP-mediated microglial process outgrowth. J. Neurosci..

[338] Eyo U.B., Peng J., Swiatkowski P., Mukherjee A., Bispo A., Wu L.J. (2014). Neuronal hyperactivity recruits microglial processes via neuronal NMDA receptors and microglial P2Y12 receptors after status epilepticus. J. Neurosci..

[339] Campagno K.E., Mitchell C.H. (2021). The P2X(7) Receptor in Microglial Cells Modulates the Endolysosomal Axis, Autophagy, and Phagocytosis. Front. Cell. Neurosci..

[340] Haynes S.E., Hollopeter G., Yang G., Kurpius D., Dailey M.E., Gan W.B., Julius D. (2006). The P2Y12 receptor regulates microglial activation by extracellular nucleotides. Nat. Neurosci..

[341] Kyrargyri V., Madry C., Rifat A., Arancibia-Carcamo I.L., Jones S.P., Chan V.T.T., Xu Y., Robaye B., Attwell D. (2020). P2Y(13) receptors regulate microglial morphology, surveillance, and resting levels of interleukin 1beta release. Glia.

[342] Illes P., Rubini P., Ulrich H., Zhao Y., Tang Y. (2020). Regulation of Microglial Functions by Purinergic Mechanisms in the Healthy and Diseased CNS. Cells.

[343] Lalo U., Palygin O., Verkhratsky A., Grant S.G., Pankratov Y. (2016). ATP from synaptic terminals and astrocytes regulates NMDA receptors and synaptic plasticity through PSD-95 multi-protein complex. Sci. Rep..

[344] Boue-Grabot E., Pankratov Y. (2017). Modulation of Central Synapses by Astrocyte-Released ATP and Postsynaptic P2X Receptors. Neural. Plast..

[345] Parkhurst C.N., Yang G., Ninan I., Savas J.N., Yates J.R., Lafaille J.J., Hempstead B.L., Littman D.R., Gan W.B. (2013). Microglia promote learning-dependent synapse formation through brain-derived neurotrophic factor. Cell.

[346] Suh H.S., Zhao M.L., Derico L., Choi N., Lee S.C. (2013). Insulin-like growth factor 1 and 2 (IGF1, IGF2) expression in human microglia: Differential regulation by inflammatory mediators. J. Neuroinflamm..

[347] Ueno M., Fujita Y., Tanaka T., Nakamura Y., Kikuta J., Ishii M., Yamashita T. (2013). Layer V cortical neurons require microglial support for survival during postnatal development. Nat. Neurosci..

[348] Masuda T., Prinz M. (2016). Microglia: A Unique Versatile Cell in the Central Nervous System. ACS Chem. Neurosci..

[349] Zhou L.J., Peng J., Xu Y.N., Zeng W.J., Zhang J., Wei X., Mai C.L., Lin Z.J., Liu Y., Murugan M. (2019). Microglia Are Indispensable for Synaptic Plasticity in the Spinal Dorsal Horn and Chronic Pain. Cell Rep..

[350] Li Y., Du X.F., Liu C.S., Wen Z.L., Du J.L. (2012). Reciprocal regulation between resting microglial dynamics and neuronal activity in vivo. Dev. Cell.

[351] Cherry J.D., Olschowka J.A., O’Banion M.K. (2014). Are “resting” microglia more “m2”?. Front. Immunol..

[352] Franco R., Fernandez-Suarez D. (2015). Alternatively activated microglia and macrophages in the central nervous system. Prog. Neurobiol..

[353] Tang Y., Le W. (2016). Differential Roles of M1 and M2 Microglia in Neurodegenerative Diseases. Mol. Neurobiol..

[354] Geloso M.C., Corvino V., Marchese E., Serrano A., Michetti F., D’Ambrosi N. (2017). The Dual Role of Microglia in ALS: Mechanisms and Therapeutic Approaches. Front. Aging Neurosci..

[355] Subramaniam S.R., Federoff H.J. (2017). Targeting Microglial Activation States as a Therapeutic Avenue in Parkinson’s Disease. Front. Aging Neurosci..

[356] Masuda T., Sankowski R., Staszewski O., Bottcher C., Amann L., Sagar G., Scheiwe C., Nessler S., Kunz P., van Loo G. (2019). Spatial and temporal heterogeneity of mouse and human microglia at single-cell resolution. Nature.

[357] Tan Y.L., Yuan Y., Tian L. (2020). Microglial regional heterogeneity and its role in the brain. Mol. Psychiatry.

[358] Harrison J.K., Jiang Y., Chen S., Xia Y., Maciejewski D., McNamara R.K., Streit W.J., Salafranca M.N., Adhikari S., Thompson D.A. (1998). Role for neuronally derived fractalkine in mediating interactions between neurons and CX3CR1-expressing microglia. Proc. Natl. Acad. Sci. USA.

[359] Ransohoff R.M., Perry V.H. (2009). Microglial physiology: Unique stimuli, specialized responses. Annu. Rev. Immunol..

[360] Veerhuis R., Nielsen H.M., Tenner A.J. (2011). Complement in the brain. Mol. Immunol..

[361] Hickman S.E., Kingery N.D., Ohsumi T.K., Borowsky M.L., Wang L.C., Means T.K., El Khoury J. (2013). The microglial sensome revealed by direct RNA sequencing. Nat. Neurosci..

[362] Lemke G. (2013). Biology of the TAM receptors. Cold Spring Harb. Perspect. Biol..

[363] Ulland T.K., Song W.M., Huang S.C., Ulrich J.D., Sergushichev A., Beatty W.L., Loboda A.A., Zhou Y., Cairns N.J., Kambal A. (2017). TREM2 Maintains Microglial Metabolic Fitness in Alzheimer’s Disease. Cell.

[364] Chhatbar C., Prinz M. (2021). The roles of microglia in viral encephalitis: From sensome to therapeutic targeting. Cell. Mol. Immunol..

[365] Farber K., Kettenmann H. (2006). Purinergic signaling and microglia. Pflügers Arch..

[366] Inoue K., Koizumi S., Tsuda M. (2007). The role of nucleotides in the neuron-glia communication responsible for the brain functions. J. Neurochem..

[367] Paolicelli R.C., Bolasco G., Pagani F., Maggi L., Scianni M., Panzanelli P., Giustetto M., Ferreira T.A., Guiducci E., Dumas L. (2011). Synaptic pruning by microglia is necessary for normal brain development. Science.

[368] Hoshiko M., Arnoux I., Avignone E., Yamamoto N., Audinat E. (2012). Deficiency of the microglial receptor CX3CR1 impairs postnatal functional development of thalamocortical synapses in the barrel cortex. J. Neurosci..

[369] Cheadle L., Rivera S.A., Phelps J.S., Ennis K.A., Stevens B., Burkly L.C., Lee W.A., Greenberg M.E. (2020). Sensory Experience Engages Microglia to Shape Neural Connectivity through a Non-Phagocytic Mechanism. Neuron.

[370] Chung W.S., Clarke L.E., Wang G.X., Stafford B.K., Sher A., Chakraborty C., Joung J., Foo L.C., Thompson A., Chen C. (2013). Astrocytes mediate synapse elimination through MEGF10 and MERTK pathways. Nature.

[371] Schaefer L. (2014). Complexity of danger: The diverse nature of damage-associated molecular patterns. J. Biol. Chem..

[372] Prinz M., Priller J. (2014). Microglia and brain macrophages in the molecular age: From origin to neuropsychiatric disease. Nat. Rev. Neurosci..

[373] Probert L. (2015). TNF and its receptors in the CNS: The essential, the desirable and the deleterious effects. Neuroscience.

[374] de Jong B.A., Huizinga T.W., Bollen E.L., Uitdehaag B.M., Bosma G.P., van Buchem M.A., Remarque E.J., Burgmans A.C., Kalkers N.F., Polman C.H. (2002). Production of IL-1β and IL-1Ra as risk factors for susceptibility and progression of relapse-onset multiple sclerosis. J. Neuroimmunol..

[375] Shastri A., Bonifati D.M., Kishore U. (2013). Innate immunity and neuroinflammation. Mediat. Inflamm..

[376] Levesque S.A., Pare A., Mailhot B., Bellver-Landete V., Kebir H., Lecuyer M.A., Alvarez J.I., Prat A., de Rivero Vaccari J.P., Keane R.W. (2016). Myeloid cell transmigration across the CNS vasculature triggers IL-1β-driven neuroinflammation during autoimmune encephalomyelitis in mice. J. Exp. Med..

[377] Rizzo F.R., Musella A., De Vito F., Fresegna D., Bullitta S., Vanni V., Guadalupi L., Stampanoni Bassi M., Buttari F., Mandolesi G. (2018). Tumor Necrosis Factor and Interleukin-1β Modulate Synaptic Plasticity during Neuroinflammation. Neural. Plast..

[378] Rovaris M., Barnes D., Woodrofe N., du Boulay G.H., Thorpe J.W., Thompson A.J., McDonald W.I., Miller D.H. (1996). Patterns of disease activity in multiple sclerosis patients: A study with quantitative gadolinium-enhanced brain MRI and cytokine measurement in different clinical subgroups. J. Neurol..

[379] Baraczka K., Pozsonyi T., Szuts I., Ormos G., Nekam K. (2003). Increased levels of tumor necrosis alpha and soluble vascular endothelial adhesion molecule-1 in the cerebrospinal fluid of patients with connective tissue diseases and multiple sclerosis. Acta Microbiol. Immunol. Hung..

[380] Hu S., Sheng W.S., Ehrlich L.C., Peterson P.K., Chao C.C. (2000). Cytokine effects on glutamate uptake by human astrocytes. Neuroimmunomodulation.

[381] Szymocha R., Akaoka H., Dutuit M., Malcus C., Didier-Bazes M., Belin M.F., Giraudon P. (2000). Human T-cell lymphotropic virus type 1-infected T lymphocytes impair catabolism and uptake of glutamate by astrocytes via Tax-1 and tumor necrosis factor alpha. J. Virol..

[382] Ohgoh M., Hanada T., Smith T., Hashimoto T., Ueno M., Yamanishi Y., Watanabe M., Nishizawa Y. (2002). Altered expression of glutamate transporters in experimental autoimmune encephalomyelitis. J. Neuroimmunol..

[383] Mitosek-Szewczyk K., Sulkowski G., Stelmasiak Z., Struzynska L. (2008). Expression of glutamate transporters GLT-1 and GLAST in different regions of rat brain during the course of experimental autoimmune encephalomyelitis. Neuroscience.

[384] Prow N.A., Irani D.N. (2008). The inflammatory cytokine, interleukin-1 beta, mediates loss of astroglial glutamate transport and drives excitotoxic motor neuron injury in the spinal cord during acute viral encephalomyelitis. J. Neurochem..

[385] Mandolesi G., De Vito F., Musella A., Gentile A., Bullitta S., Fresegna D., Sepman H., Di Sanza C., Haji N., Mori F. (2017). miR-142-3p Is a Key Regulator of IL-1β-Dependent Synaptopathy in Neuroinflammation. J. Neurosci..

[386] Hardingham G.E., Bading H. (2010). Synaptic versus extrasynaptic NMDA receptor signalling: Implications for neurodegenerative disorders. Nat. Rev. Neurosci..

[387] Hardingham G.E. (2009). Coupling of the NMDA receptor to neuroprotective and neurodestructive events. Biochem. Soc. Trans..

[388] Hardingham G.E., Fukunaga Y., Bading H. (2002). Extrasynaptic NMDARs oppose synaptic NMDARs by triggering CREB shut-off and cell death pathways. Nat. Neurosci..

[389] Yan J., Bengtson C.P., Buchthal B., Hagenston A.M., Bading H. (2020). Coupling of NMDA receptors and TRPM4 guides discovery of unconventional neuroprotectants. Science.

[390] Takaki J., Fujimori K., Miura M., Suzuki T., Sekino Y., Sato K. (2012). L-glutamate released from activated microglia downregulates astrocytic L-glutamate transporter expression in neuroinflammation: The ‘collusion’ hypothesis for increased extracellular L-glutamate concentration in neuroinflammation. J. Neuroinflamm..

[391] Yu Z., Cheng G., Wen X., Wu G.D., Lee W.T., Pleasure D. (2002). Tumor necrosis factor alpha increases neuronal vulnerability to excitotoxic necrosis by inducing expression of the AMPA-glutamate receptor subunit GluR1 via an acid sphingomyelinase- and NF-kappaB-dependent mechanism. Neurobiol. Dis..

[392] Leonoudakis D., Zhao P., Beattie E.C. (2008). Rapid tumor necrosis factor alpha-induced exocytosis of glutamate receptor 2-lacking AMPA receptors to extrasynaptic plasma membrane potentiates excitotoxicity. J. Neurosci..

[393] Ogoshi F., Yin H.Z., Kuppumbatti Y., Song B., Amindari S., Weiss J.H. (2005). Tumor necrosis-factor-alpha (TNF-α) induces rapid insertion of Ca^2+^-permeable alpha-amino-3-hydroxyl-5-methyl-4-isoxazole-propionate (AMPA)/kainate (Ca-A/K) channels in a subset of hippocampal pyramidal neurons. Exp. Neurol..

[394] Ferguson A.R., Christensen R.N., Gensel J.C., Miller B.A., Sun F., Beattie E.C., Bresnahan J.C., Beattie M.S. (2008). Cell death after spinal cord injury is exacerbated by rapid TNF alpha-induced trafficking of GluR2-lacking AMPARs to the plasma membrane. J. Neurosci..

[395] Bolton C., Paul C. (1997). MK-801 limits neurovascular dysfunction during experimental allergic encephalomyelitis. J. Pharmacol. Exp. Ther..

[396] Paul C., Bolton C. (2002). Modulation of blood-brain barrier dysfunction and neurological deficits during acute experimental allergic encephalomyelitis by the N-methyl-D-aspartate receptor antagonist memantine. J. Pharmacol. Exp. Ther..

[397] Wheeler D., Knapp E., Bandaru V.V., Wang Y., Knorr D., Poirier C., Mattson M.P., Geiger J.D., Haughey N.J. (2009). Tumor necrosis factor-alpha-induced neutral sphingomyelinase-2 modulates synaptic plasticity by controlling the membrane insertion of NMDA receptors. J. Neurochem..

[398] Grasselli G., Rossi S., Musella A., Gentile A., Loizzo S., Muzio L., Di Sanza C., Errico F., Musumeci G., Haji N. (2013). Abnormal NMDA receptor function exacerbates experimental autoimmune encephalomyelitis. Br. J. Pharmacol..

[399] Sulkowski G., Dabrowska-Bouta B., Chalimoniuk M., Struzynska L. (2013). Effects of antagonists of glutamate receptors on pro-inflammatory cytokines in the brain cortex of rats subjected to experimental autoimmune encephalomyelitis. J. Neuroimmunol..

[400] Bolton C., Wood E.G., Ayoub S.S. (2013). N-Methyl-D-aspartate (NMDA) receptor involvement in central nervous system prostaglandin production during the relapse phase of chronic relapsing experimental autoimmune encephalomyelitis (CR EAE). Fundam. Clin. Pharmacol..

[401] Suhs K.W., Fairless R., Williams S.K., Heine K., Cavalie A., Diem R. (2014). N-methyl-D-aspartate receptor blockade is neuroprotective in experimental autoimmune optic neuritis. J. Neuropathol. Exp. Neurol..

[402] Dabrowska-Bouta B., Struzynska L., Chalimoniuk M., Frontczak-Baniewicz M., Sulkowski G. (2015). The influence of glutamatergic receptor antagonists on biochemical and ultrastructural changes in myelin membranes of rats subjected to experimental autoimmune encephalomyelitis. Folia Neuropathol..

[403] Fairless R., Bading H., Diem R. (2021). Pathophysiological Ionotropic Glutamate Signalling in Neuroinflammatory Disease as a Therapeutic Target. Front. Neurosci..

[404] Floden A.M., Li S., Combs C.K. (2005). Beta-amyloid-stimulated microglia induce neuron death via synergistic stimulation of tumor necrosis factor alpha and NMDA receptors. J. Neurosci..

[405] Han P., Whelan P.J. (2010). Tumor necrosis factor alpha enhances glutamatergic transmission onto spinal motoneurons. J. Neurotrauma..

[406] Rossi S., Muzio L., De Chiara V., Grasselli G., Musella A., Musumeci G., Mandolesi G., De Ceglia R., Maida S., Biffi E. (2011). Impaired striatal GABA transmission in experimental autoimmune encephalomyelitis. Brain Behav. Immun..

[407] Mandolesi G., Grasselli G., Musella A., Gentile A., Musumeci G., Sepman H., Haji N., Fresegna D., Bernardi G., Centonze D. (2012). GABAergic signaling and connectivity on Purkinje cells are impaired in experimental autoimmune encephalomyelitis. Neurobiol. Dis..

[408] Pribiag H., Stellwagen D. (2013). TNF-α downregulates inhibitory neurotransmission through protein phosphatase 1-dependent trafficking of GABA(A) receptors. J. Neurosci..

[409] Nistico R., Mango D., Mandolesi G., Piccinin S., Berretta N., Pignatelli M., Feligioni M., Musella A., Gentile A., Mori F. (2013). Inflammation subverts hippocampal synaptic plasticity in experimental multiple sclerosis. PLoS ONE.

[410] Wang D.S., Zurek A.A., Lecker I., Yu J., Abramian A.M., Avramescu S., Davies P.A., Moss S.J., Lu W.Y., Orser B.A. (2012). Memory deficits induced by inflammation are regulated by alpha5-subunit-containing GABAA receptors. Cell Rep..

[411] Yan X., Jiang E., Weng H.R. (2015). Activation of toll like receptor 4 attenuates GABA synthesis and postsynaptic GABA receptor activities in the spinal dorsal horn via releasing interleukin-1 beta. J. Neuroinflamm..

[412] Roseti C., van Vliet E.A., Cifelli P., Ruffolo G., Baayen J.C., Di Castro M.A., Bertollini C., Limatola C., Aronica E., Vezzani A. (2015). GABAA currents are decreased by IL-1beta in epileptogenic tissue of patients with temporal lobe epilepsy: Implications for ictogenesis. Neurobiol. Dis..

[413] Paul A.M., Branton W.G., Walsh J.G., Polyak M.J., Lu J.Q., Baker G.B., Power C. (2014). GABA transport and neuroinflammation are coupled in multiple sclerosis: Regulation of the GABA transporter-2 by ganaxolone. Neuroscience.

[414] Su J., Yin J., Qin W., Sha S., Xu J., Jiang C. (2015). Role for pro-inflammatory cytokines in regulating expression of GABA transporter type 1 and 3 in specific brain regions of kainic acid-induced status epilepticus. Neurochem. Res..

[415] Fu C.Y., He X.Y., Li X.F., Zhang X., Huang Z.W., Li J., Chen M., Duan C.Z. (2015). Nefiracetam Attenuates Pro-Inflammatory Cytokines and GABA Transporter in Specific Brain Regions of Rats with Post-Ischemic Seizures. Cell. Physiol. Biochem..

[416] Cawley N., Solanky B.S., Muhlert N., Tur C., Edden R.A., Wheeler-Kingshott C.A., Miller D.H., Thompson A.J., Ciccarelli O. (2015). Reduced gamma-aminobutyric acid concentration is associated with physical disability in progressive multiple sclerosis. Brain.

[417] Takeuchi H., Jin S., Suzuki H., Doi Y., Liang J., Kawanokuchi J., Mizuno T., Sawada M., Suzumura A. (2008). Blockade of microglial glutamate release protects against ischemic brain injury. Exp. Neurol..

[418] Takeuchi H., Jin S., Wang J., Zhang G., Kawanokuchi J., Kuno R., Sonobe Y., Mizuno T., Suzumura A. (2006). Tumor necrosis factor-alpha induces neurotoxicity via glutamate release from hemichannels of activated microglia in an autocrine manner. J. Biol. Chem..

[419] Haroon E., Miller A.H., Sanacora G. (2017). Inflammation, Glutamate, and Glia: A Trio of Trouble in Mood Disorders. Neuropsychopharmacology.

[420] Sicotte N.L., Kern K.C., Giesser B.S., Arshanapalli A., Schultz A., Montag M., Wang H., Bookheimer S.Y. (2008). Regional hippocampal atrophy in multiple sclerosis. Brain.

[421] Geurts J.J., Calabrese M., Fisher E., Rudick R.A. (2012). Measurement and clinical effect of grey matter pathology in multiple sclerosis. Lancet Neurol..

[422] Colasanti A., Guo Q., Giannetti P., Wall M.B., Newbould R.D., Bishop C., Onega M., Nicholas R., Ciccarelli O., Muraro P.A. (2016). Hippocampal Neuroinflammation, Functional Connectivity, and Depressive Symptoms in Multiple Sclerosis. Biol. Psychiatry.

[423] Chiaravalloti N.D., DeLuca J. (2008). Cognitive impairment in multiple sclerosis. Lancet Neurol..

[424] Ziehn M.O., Avedisian A.A., Tiwari-Woodruff S., Voskuhl R.R. (2010). Hippocampal CA1 atrophy and synaptic loss during experimental autoimmune encephalomyelitis, EAE. Lab. Investig..

[425] Prochnow N., Gold R., Haghikia A. (2013). An electrophysiologic approach to quantify impaired synaptic transmission and plasticity in experimental autoimmune encephalomyelitis. J. Neuroimmunol..

[426] Planche V., Panatier A., Hiba B., Ducourneau E.G., Raffard G., Dubourdieu N., Maitre M., Leste-Lasserre T., Brochet B., Dousset V. (2017). Selective dentate gyrus disruption causes memory impairment at the early stage of experimental multiple sclerosis. Brain Behav. Immun..

[427] Crombe A., Planche V., Raffard G., Bourel J., Dubourdieu N., Panatier A., Fukutomi H., Dousset V., Oliet S., Hiba B. (2018). Deciphering the microstructure of hippocampal subfields with in vivo DTI and NODDI: Applications to experimental multiple sclerosis. Neuroimage.

[428] Rocca M.A., Barkhof F., De Luca J., Frisen J., Geurts J.J.G., Hulst H.E., Sastre-Garriga J., Filippi M., Group M.S. (2018). The hippocampus in multiple sclerosis. Lancet Neurol..

[429] Di Filippo M., Portaccio E., Mancini A., Calabresi P. (2018). Multiple sclerosis and cognition: Synaptic failure and network dysfunction. Nat. Rev. Neurosci..

[430] Heppner F.L., Greter M., Marino D., Falsig J., Raivich G., Hovelmeyer N., Waisman A., Rulicke T., Prinz M., Priller J. (2005). Experimental autoimmune encephalomyelitis repressed by microglial paralysis. Nat. Med..

[431] Patsopoulos N.A., Baranzini S.E., Santaniello A., Shoostari P., Cotsapas C., Wong G., Beecham A.H., James T., Replogle J., Vlachos I.S. (2019). Multiple sclerosis genomic map implicates peripheral immune cells and microglia in susceptibility. Science.

[432] Guerrero B.L., Sicotte N.L. (2020). Microglia in Multiple Sclerosis: Friend or Foe?. Front. Immunol..

